# Immune-checkpoint-inhibitor therapy directed against PD-L1 is tolerated in the heart without manifestation of cardiac inflammation in a preclinical reversible melanoma mouse model

**DOI:** 10.3389/fmmed.2024.1487526

**Published:** 2025-01-06

**Authors:** Caroline Schoenherr, Stefan Pietzsch, Cristina Barca, Franziska E. Müller, Frauke S. Bahr, Martina Kasten, Andre Zeug, Sergej Erschow, Christine S. Falk, Evgeni Ponimaskin, James T. Thackeray, Denise Hilfiker-Kleiner, Melanie Ricke-Hoch

**Affiliations:** ^1^ Department of Cardiology and Angiology, Hannover Medical School, Hannover, Germany; ^2^ Department of Hematology, Hemostasis, Oncology and Stem Cell Transplantation, Hannover Medical School, Hannover, Germany; ^3^ Department of Human Genetics, Hannover Medical School, Hannover, Germany; ^4^ Department of Nuclear Medicine, Hannover Medical School, Hannover, Germany; ^5^ Department of Cellular Neurophysiology, Hannover Medical School, Hannover, Germany; ^6^ Institute of Transplant Immunology, IFB-Tx, Hannover Medical School, Hannover, Germany; ^7^ Department of Cardiovascular Complications of Oncologic Therapies, Medical Faculty of the Philipps University Marburg, Marburg, Germany

**Keywords:** immune checkpoint inhibitors, PD-L1, inflammation, cancer, cardio-oncology, cardiotoxicity

## Abstract

Immune-checkpoint-inhibitors (ICI) target key regulators of the immune system expressed by cancer cells that mask those from recognition by the immune system. They have improved the outcome for patients with various cancer types, such as melanoma. ICI-based therapy is frequently accompanied by immune-related adverse side effects (IRAEs). The reversible melanoma cancer mouse model (B16F10 cells stably expressing a ganciclovir (GCV)-inducible suicide gene in C57BL/6N mice: B16F10-GCV) allows chemotherapy-free tumor elimination in advanced disease stage and demonstrates almost complete recovery of the mouse heart from cancer-induced atrophy, molecular impairment and heart failure. Thus, enabling the study of anti-cancer-therapy effects. Here, we analyzed potential cardiac side effects of antibody-mediated PD-L1 inhibition in the preclinical B16F10-GCV mouse model after tumor elimination and 2 weeks recovery (50 days after tumor inoculation). Anti-PD-L1 treatment was associated with improved survival as compared to isotype control (Ctrl) treated mice. Surviving anti-PD-L1 and Ctrl mice showed similar cardiac function, dimensions and the expression of cardiac stress and hypertrophy markers. Although anti-PD-L1 treatment was associated with increased troponin I type 3 cardiac (TNNI3) blood levels, cardiac mRNA expression of macrophage markers and elevated cardiac levels of secreted inflammatory factors compared to Ctrl treatment, both groups showed a comparable density of inflammatory cells in the heart (using CXCR4-ligand ^68^Ga-Pentixafor in PET-CT and immunohistochemistry). Thus, anti-PD-L1 therapy improved survival in mice with advanced melanoma cancer with no major cardiac phenotype or inflammation 50 days after tumor inoculation. Without a second hit that triggers the inflammatory response, anti-PD-L1 treatment appears to be safe for the heart in the preclinical melanoma mouse model.

## Introduction

The development and clinical use of immune checkpoint inhibitors (ICIs) have recently improved the survival of patients with various types of cancer, including malignant melanoma, lung cancer, kidney cancer and others (Palaskas et al., 2020; Mahoney et al., 2015). In contrast to conventional chemotherapy or targeted therapies that mostly have a direct effect on cancer cells, ICIs mediate their effects indirectly by enabling the immune system to recognize cancer cells mainly by tumor-specific, cytotoxic T lymphocytes. Within the group of ICIs, there exist different “generations” of drugs, each of which differ in its target molecule and thus intervene at different points of entry in the immune system, but all have in common the overarching effect of increased sensitization of the endogenous immune defense mechanisms (Varricchi et al., 2017). An immunosuppressive effect of so-called immune checkpoints is exerted by expression and mutual recognition of various cell surface markers (cytotoxic T-lymphocyte associated protein 4 (CTLA-4), programmed cell death 1 (PD-1), programmed cell death ligand 1 (PD-L1)) on T cells and antigen-presenting cells (de Mello et al., 2017). This effect protects against an “overshoot” of the immune response or autoimmune diseases during antigen recognition and subsequent T cell activation. High expression of these surface markers enables tumor cells to evade recognition and thus elimination by the immune system (de Mello et al., 2017). The blockade of this ligand receptor interaction using ICIs prevents the following immunosuppressive effect and the sensitized immune system reacts with an intensified immune response, which is now also directed against tumor cells that previously remained undetected.

An intervention in such central mechanisms of the immune response can be associated with so-called immune-related adverse events (IRAE) (Varricchi et al., 2017). Initially, the most common mild side effects observed with various ICIs were rash, fatigue, pruritus or diarrhea (Varricchi et al., 2017). Recently, increasing evidence has been reported for acute and sometimes life-threatening myocarditis related to ICI treatment (Neilan et al., 2018). Due to the acute form of ICI-associated cardiotoxicity and the demonstrated expression of PD-1/PD-L1 in the heart, this signaling pathway is assumed to be of particular importance in the development of ICI-induced myocarditis (Neilan et al., 2018). The release of heart muscle-specific proteins (cardiac antigens) due to severe cardiac atrophy, e.g., in tumor disease, could lead to sensitization to cardiac inflammation caused by ICI. FDA approved medications for PD-L1 blockade include humanized monoclonal antibodies such as Atezolizumab, Durvalumab, Avelumab (Schardt, 2020).

Previous clinical studies on cardiotoxicity frequently use healthy animal models that do not adequately capture the clinical situation of cancer diseased patients, since cancer itself is able to impact on the cardiovascular system, such as cardiac atrophy and inflammation, alterations in mitochondrial oxidative characteristics and metabolism, and a functional decline (Pietzsch et al., 2021; Salloum et al., 2023). In addition, the systemic impact of malignant tumor diseases, including the release of cytokines and metabolites, impairs several cardioprotective signaling pathways. Therefore, the effect of anti-cancer medications on the heart may be different in healthy and in cancer diseased mice. As mouse cancer models may exhibit high mortality complicating longterm studies, we established a reversible B16F10 melanoma mouse model that was stably transduced with a suicide gene inducing cell death upon exposure to ganciclovir (GCV, model named as B16F10-GCV mice) (Pincha et al., 2011; Pietzsch et al., 2021). In B16F10-GCV mice, we have previously demonstrated almost complete cardiac recovery from cancer-induced atrophy, molecular impairment and heart failure (Pietzsch et al., 2021). In brief, during advanced cancer stage, B16F10-GCV mice showed massive cardiac atrophy related to cardiomyocyte atrophy (without the induction of cardiomyocyte apoptosis), and impaired cardiac function. Cardiac atrophy and dysfunction were partly caused by an imbalance of cardiac protein synthesis and degradation related to alterations in systemic and cardiac metabolism such as an impairment in insulin signaling (Thackeray et al., 2017; Pietzsch et al., 2021). After recovery from melanoma cancer, B16F10-GCV mice displayed functional, metabolic and morphological cardiac recovery (Pietzsch et al., 2021). Thus, this model enables investigation of longlasting effects of cancer on the cardiovascular system after chemotherapy-free tumor elimination, and to differentiate them from side effects of anti-cancer therapies (Pietzsch et al., 2021). In this context, we were already able to demonstrate that doxorubicin treatment during the advanced cancer disease stage is associated with increased mortality of B16F10-GCV mice. The surviving mice revealed morphological and functional cardiac recovery but showed longlasting alterations in the cardiac gene expression profile, especially in the circadian rhythm pathway, which is a known mediator of the DNA damage response pathway (DDR) and might contribute to late cardiotoxicity. In the present study, we applied a mouse-specific monoclonal antibody treatment regime targeting PD-L1 to investigate potential side effects of ICI therapy on the heart after recovery from tumor disease in the reversible B16F10 melanoma mouse model. Since in cancer patients with ICI therapy IRAEs often occur within a timeframe of approximately 6 weeks after ICI therapy, we performed the final analysis for our study 50 days after tumor induction and 43 days after starting anti-PD-L1 therapy. To detect possible inflammatory response during anti-PD-L1 therapy, serial ^68^Ga-Pentixafor PET-CT measurements were done. The aim was to analyze whether a PD-L1-directed ICI therapy induces cardiac IRAE after recovery from melanoma cancer disease. An elevated immune response after recovery from cancer disease might therefore contribute to late autoimmune side effects such as myocarditis.

## Methods

### B16F10 melanoma cells

Murine melanoma cell line B16F10 was obtained from ATCC. Cells were grown in DMEM culture medium containing 4.5 g/L glucose (Capricorn) supplemented with 10% FCS (Biochrom AG) and penicillin/streptomycin (100 U/100 µg per ml, Gibco). For injection, tumor cells were grown to confluence and detached from cell culture flasks by treatment with 0.25% trypsin/EDTA (Gibco). Cell pellet was washed twice with prewarmed, sterile PBS; then, cells were suspended in PBS and counted. As previously described, B16F10 cells were genetically modified by lentiviral transduction with a herpes simplex virus type 1 thymidine kinase (HSVtk), firefly luciferase and yellow fluorescent protein (YFP) containing construct (Pincha et al., 2011; Pietzsch et al., 2021). Single cell clones were isolated by limiting dilution and checked for positive YFP signal in FACS. A fully YFP-positive cell clone (B16F10HSVtk-c) was used for all *in vivo* experiments. HSVtk1 enabling cell-specific induction of apoptosis was activated by addition of GCV as described (Tomicic et al., 2002).

### Animal experiments–B16F10 melanoma mouse model

**FIGURE 1:**
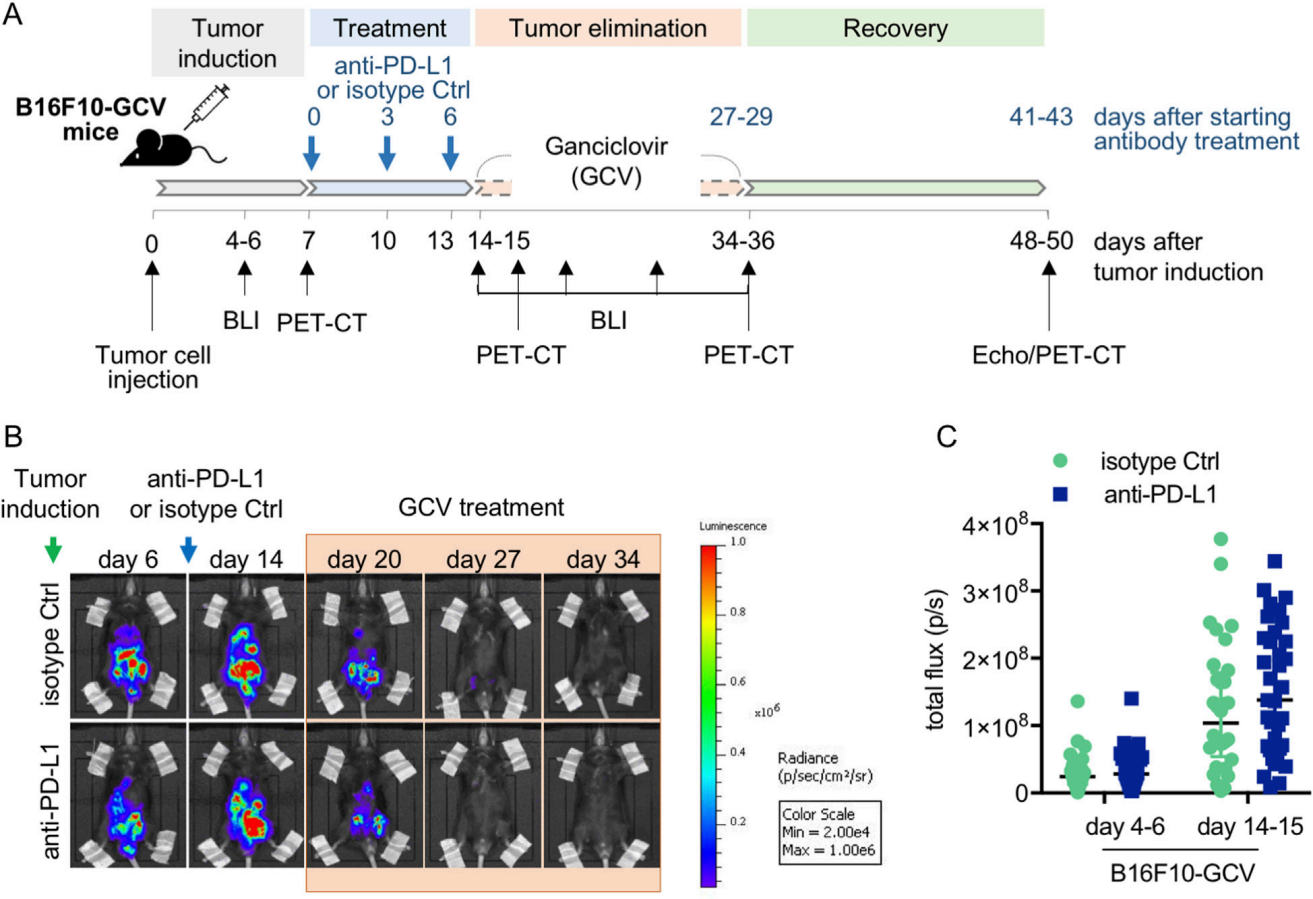
Anti-PD-L1 treatment in B16F10-GCV mice. **(A)** Scheme for anti-PD-L1 or isotype Ctrl treatment in B16F10-GCV mice. **(B)** Serial BLI of B16F10-GCV mice following tumor inoculation of luciferase and HSVtk expressing B16F10 cells. Mice were monitored up to 50 days after tumor inoculation and **(C)** quantitative analysis of the tumor burden was performed by measurement of the total flux (p/s) on day 4–6 (pre PD-L1 inhibition) and day 14–15 (post PD-L1 inhibition) after tumor inoculation, anti-PD-L1 (N = 35) or isoptype Ctrl (Ctrl) (N = 32). Data were presented as median and IQR, n.s vs. B16F10-GCV isoptype Ctrl, Mann-Whitney *U* test.

As described before, male C57BL6/N mice (12 ± 2 weeks of age, Charles River Germany) were injected intraperitoneally (i.p.) with B16F10HSVtk-c melanoma cells (B16F10 mice, 1 × 10^6^ cells) or PBS as a vehicle (Thackeray et al., 2017; Pietzsch et al., 2021). Single doses of 100 μg monoclonal antibody per animal against PD-L1 (GolnVivo™ purified anti-mouse CD274 antibody clone 10F.9G2, no 124328, BioLegend) or corresponding isotype control (Ctrl, GolnVivo purified rat IgG2b,k isotype Ctrl, no 400666, BioLegend) were applied i.p. at day 7, 10, and 13 after tumor cell or PBS injection for a total of 3 doses (Figure 1A, Supplementary Figure S1A). Treatment with GCV (80 mg/kg BW twice daily) was started at advanced tumor disease stage at day 14 based on the guidelines of recognition of distress in experimental animals proposed by Morton and Griffith (Figure 1A) (Morton and Griffiths, 1985). Using the same protocol, GCV treatment of cancer-free control mice (GCV mice) was started at day 14 after PBS vehicle injection (Supplementary Figure S1A). GCV treatment was continued until day 34–36 when signal loss in IVIS B16F10-GCV mice occurred. For live visualization of B16F10 melanoma cells, mice were imaged using an *in vivo* imaging system (IVIS) Lumina II (Caliper Life Sciences) for 1 min following i.p. injection of 0.8 mg/mouse D-Luciferin (AppliChem). Whole animal imaging was performed from a ventral perspective. Bioluminescene radiance was analyzed using Living Image (BLI) 4.0 software. After melanoma cell inoculation, B16F10-GCV and GCV mice received continuous analgesia (Novalgin, 500 mg/mL in drinking water). Mice were housed in groups of five and maintained on a 14 h/10 h light/dark cycle with standard laboratory chow and water freely available. Animal health condition was assessed based on the guidelines of recognition of distress in experimental animals as proposed by Morton and Griffith (Morton and Griffiths, 1985).

All animal studies were in accordance with the German animal welfare legislation and with the European Communities Council Directive 86/609/EEC and 2010/63/EU to protect animals used for experimental purposes under consideration of the ARRIVE guidelines. Furthermore, the experiments were approved by the local Institutional Animal Care and Research Advisory Committee and permitted by the local authority.

### Echocardiography

Echocardiography was performed on anesthetized mice as described previously (Thackeray et al., 2017; Pietzsch et al., 2021). In brief, contractile function and heart rate were assessed by echocardiography using the Vevo 3100 system (VisualSonics) after tumor elimination and 2 weeks recovery (day 50 ± 3 after tumor cell injection). Anesthesia was induced with 4% (in 100% oxygen) isoflurane, followed by maintenance at 0.5%–1% isoflurane via a special vaporizer for rodents delivered by a small nose cone (VisualSonics). For echocardiographic image acquisition, the animal was placed in a supine position on a prewarmed platform and the body temperature was maintained at 37°C during the entire procedure. Echocardiographic measurements were obtained 5 min following the induction of anesthetic when heart rate had stably recovered to exclude the variation in cardiac function created by time after induction. Parasternal short- or long-axis views were recorded in B- and M-mode at the level of the papillary muscle, and still images were used to measure LV end-diastolic diameter (LVEDD) and LV end-systolic diameter (LVESD) and calculate fractional shortening ([LVEDD–LVESD]/LVEDD x 100). Cardiac output was calculated by VisualSonics Vevo 3100 software version 3.

### Serial ^68^Ga-Pentixafor PET-CT imaging protocol

As previously described, ^68^Ga-Pentixafor was synthesized by using an automated module and CPCR4.2 precursor provided by Scintomics GmbH (Fürstenfeldbruck, Germany) (Thackeray et al., 2015). ^18^F-FDG (fluorodeoxyglucose) was synthesized using standard kits and production methods. PET was performed using a dedicated small animal system (Inveon DPET, Siemens, Knoxville) as reported before (Thackeray et al., 2015). Animals underwent serial imaging using the CXCR4-ligand ^68^Ga-Pentixafor measured in PET-CT imaging at day 7, day 15, day 35 and day 49 after tumor cell inoculation (day 0, day 8, day 28 and day 42 after starting anti-PD-L1 or isotype Ctrl treatment) to identify tissue inflammation (Figure 1A). In brief, anesthetized animals (1.5% isoflurane, 0.6 L/min O_2_) were positioned prone on the imaging bed (Minerve) with the heart centered in the scanner field of view. ^68^Ga-Pentixafor (12.6 ± 1.3 MBq) was injected intravenously as 0.10 mL bolus via the tail vein. After conscious tracer distribution, a 10 min static listmode scan was acquired at 50 min after injection under isoflurane anesthesia. Afterwards, 0.2 mL ^18^F-FDG (20 ± 2 MBq) was administrated i.p. under anesthesia to enable accurate localization of the myocardium. After 20 min under continued isoflurane, a second 10 min static scan was performed (Glasenapp et al., 2021).

All PET images were reconstructed to a 128 × 128 × 159 image matrix (0.78 × 0.78 × 0.80 mm) using an OSEM3D/MAP (ordered subset expectation maximization/maximum *a posteriori*) algorithm (β = 0.01, 2 OSEM iterations, 18 MAP iterations) with scanner-applied scatter correction. A ^57^Co transmission scan (10 min) was used for attenuation correction (Thackeray et al., 2015). Image analysis was performed with Inveon Research Workplace 4.2 (Siemens Medical Solutions). First, ^18^F-FDG images were used to manually create regions of interest for LV myocardium by interactive thresholding as previously described (Thackeray et al., 2015). These regions of interest were imported onto co-registered ^68^Ga-pentixafor images to calculate ^68^Ga-pentixafor uptake. The signal was semiquantitatively analyzed as percent injected dose per gram of tissue (%ID/g).

## Murine plasma measurements

Murine plasma samples were acquired by 10 min centrifugation at 1,500 rpm of right ventricular blood in ethylenediaminetetraacetic acid (EDTA) containing vials and stored at −80°C. Mouse troponin I type 3 cardiac (TNNI3; Novus Biologicals #NBP3-00456) levels were measured in plasma using a commercial ELISA kit following the manufacturer’s protocol. Concentrations were detected with a Thermo Scientific Varioskan Flash plate reader using SkanIt Software 2.4.5 (Thermo Scientific).

### Lipid peroxidation (malondialdehyde, MDA) detection

Lipid peroxidation (nmol/mg protein) were measured in LV tissue lysates using a commercial MDA assay kit (Abcam ab118970) respectively, according to the manufacturer’s protocol. Concentrations were detected with a Thermo Scientific Varioskan Flash plate reader using SkanIt Software 2.4.5 (Thermo Scientific).

### Histology and immunostaining

For cardiac morphological analyses, hearts were perfused, embedded in OCT Tissue-Tek and frozen at −80°C. Cardiac cryosections were stained with H&E as described (Hilfiker-Kleiner et al., 2004). Cardiomyocyte cross-sectional area (CSA) was determined on longitudinal cardiac sections of B16F10-GCV mice after staining with Fluorescein-labelled wheat germ agglutinin (WGA; Vector Laboratories Inc., FL-1021) and Hoechst 33258 (SIGMA-Aldrich) as previously described. At least 30–50 cells per individual heart were measured (Heimerl et al., 2020). Interstitial collagen was analysed in picro-Sirius red F3BA-stained LV cryosections (Hilfiker-Kleiner et al., 2004). Global inflammation was stained in LV cryosections with the pan-inflammatory marker CD45 (BD Pharmingen, Clone 30-F11, #550539) counterstained with eosin as described (Hoch et al., 2011). The average number of CD45^+^ cells was determined per field (5 fields/section and 3 sections/animal). HE, Sirius Red and CD45 staining images were acquired by bright field microscopy using Axio Observer 7 and Zen 2.6 pro software (Carl Zeiss Jena).

Inflammation was stained in LV cryosections with antibodies recognising CD68 (abcam ab53444), CD80 (abcam ab106162), CD206 (R&D Systems AF2535) and CD8a (Cell Signaling Technologies #98941) counterstained with Hoechst 33258 (SIGMA-Aldrich). Cardiac slices were imaged on a Zeiss LSM780 with a LD C-Apochromat 40x/1.2 W objective and Zen2013 imaging software in online-fingerprinting mode with pre-defined spectra for each fluorescent protein obtained from single stainings. Five regions per slice were randomly chosen along the sagittal axis and z-stacks were acquired with 12 planes in 0.5 µm distance and a pixel size of 0.208 µm (1024 × 1024 pixels, pixel dwell time 1.57 µs). Image analysis was done in maximum-intensity projections using ImageJ and Matlab (Mathworks). Mean fluorescence intensities were measured using ImageJ ‘Measure’ tool for each individual channel in each of the five imaged regions. A mean of the five regions was taken to represent the mean fluorescence per animal.

Number of macrophages was counted manually using ImageJ “CellCounter” Plugin in a merged image of CD68, CD80 and CD206 fluorescence signal in each of the five imaged regions. Cells were only counted when the macrophage marker signal could be assigned to a Hoechst-stained nucleus. A mean of the five regions was used to display the mean number of macrophages per animal per field of view. Pearson’s correlation coefficient was calculated using custom-written Matlab scripts for each of the five regions per animal. Analysis strategy follows the colocalization theory of Scientific Volume Imaging (https://svi.nl/ColocalizationTheory).

Negative control for the correlation coefficient was obtained for each image by rotation of the second channel by 90°C and 50% pixel shift followed by the same correlation analysis as for the original image.

### Multiplex assays of cardiac murine tissue

For multiplex analysis, samples from LV tissue of anti-PD-L1 and isotype Ctrl treated mice were generated with the Bio-Plex^®^ cell lysis kit (#171304011, Bio-Rad) according to the manufacturer’s protocol. The protein concentration of the LV lysates was measured with Bradford reagent (Bio-Rad) and LV lysates were diluted with Bio-Plex sample diluent to a protein contration of 1 mg/mL. The Bio-Plex Pro™ Mouse Cytokine 23-plex Assay (M60009RDPD, Bio-Rad) was used as recommended by the manufacturer and measured with the Bio-Plex 200 system (Bio-Rad).

### RNA isolation, cDNA synthesis and qRT-PCR

Total RNA from adult murine hearts was isolated with TRIzol^®^ Reagent (Life technologies) in accordance with the manufacturer’s instructions. cDNA synthesis using Superscript III (Invitrogen), 2 μg of total RNA and random hexamer primers (SIGMA-Aldrich) was performed according to the manufacturer’s protocols as previously described (Heimerl et al., 2020). Semi-quantitative real-time PCR using the SYBR green dye method (SYBR Green qPCR 2xMastermix-Kit, Thermo Fisher Scientific) was performed with the AriaMX Real-Time PCR System (Agilent Technologies). Sequences of qRT-PCR primers used in this study are provided below. mRNA expression levels were normalised using the 2^−ΔΔCT^ method relative to 18S.

**Table:** Sequences of qRT-PCR primers

mRNA	Sense primers (5′ to 3′)	Antisense primers (5′ to 3′)
mmu *18S*	GTA​ACC​CGT​TGA​ACC​CCA​TT	CCA​TCC​AAT​CGG​TAG​TAG​CG
mmu *Adcy5*	GAA​CTG​CCA​GCT​TCG​GAG​AG	CAG​CCC​CGA​GGT​GAG​AAG​TA
mmu *Adcy6*	TGG​GGT​TTG​ACG​ACA​CTG​AG	GCA​GAG​CGG​AAC​TGC​TTA​GA
mmu *Adrge1*	GAG​ACA​TCC​ACT​CTG​GGC​AC	GGG​GCC​CCT​GTA​GAT​ACT​GA
mmu *Anp*	GCC​GGT​AGA​AGA​TGA​GGT​CA	GGG​CTC​CAA​TCC​TGT​CAA​TC
mmu *Atp2a2*	CAA​ACC​AGA​TGT​CCG​TGT​GC	TGA​TGG​CAC​TTC​ACT​GGC​TT
mmu *Bnp*	ATC​CGA​TCC​GGT​CTA​TCT​TG	CCA​GTC​TCC​AGA​GCA​ATT​CA
mmu *Cd4*	GCA​AAG​TCT​CGA​GCC​CTC​AT	GCA​CAT​GGT​GGT​CTC​CTT​GA
mmu *Cd8a*	GGA​TTG​GAC​TTC​GCC​TGT​GA	TGG​GAC​ATT​TGC​AAA​CAC​GC
mmu *Cd19*	TCA​TTG​CAA​GGT​CAG​CAG​TGT​G	GGG​TCA​GTC​ATT​CGC​TTC​CTT
mmu *Cd38*	ACT​GGA​GAG​CCT​ACC​ACG​AA	AGT​GGG​GCG​TAG​TCT​TCT​CT
mmu *Cd80*	TTT​CAG​ACC​GGG​GCA​CAT​AC	AGA​AGC​GAG​GCT​TTG​GGA​AA
mmu *Cd206*	GAT​GAC​CTG​TGC​TCG​AGA​GG	TCG​CTT​CCC​TCA​AAG​TGC​AA
mmu *Col1a1*	ACA​GAC​GAA​CAA​CCC​AAA​CT	GGT​TTT​TGG​TCA​CGT​TCA​GT
mmu *Il-1β*	GCC​CAT​CCT​CTG​TGA​CTC​AT	AGG​CCA​CAG​GTA​TTT​TGT​CG
mmu *αMHC*	GGA​AGA​GCG​AGC​GGC​GCA​TCA​AGG	GTC​TGC​TGG​AGA​GGT​TAT​TCC​TCG
mmu *βMHC*	CAAGTTCCGCAAGGTGC	AAA​TTG​CTT​TAT​TCT​GCT​TCC​AC
mmu *MnSOD*	ACC​TGC​CTT​ACG​ACT​ATG​GC	AGC​CTG​AAC​CTT​GGA​CTC​C
mmu *Pd-l1*	GGC​AGG​AGA​GGA​GGA​CCT​TA	TGC​AGC​TTG​ACG​TCT​GTG​AT
mmu *Ryr2*	CATGACCAACCCTGTCCCTGTC	CTT​CCG​GCT​CCC​CAT​AGC​G
mmu *Tnfα*	GGT​GCC​TAT​GTC​TCA​GCC​TCT​T	GCC​ATA​GAA​CTG​ATG​AGA​GGG​AG

### Protein isolation, SDS-PAGE and Western blot

Protein expression levels were determined by Western blotting, using SDS-PAGE as described (Heimerl et al., 2020). In brief, total protein was isolated by lysing frozen LV tissue in RIPA buffer supplemented with 10 μM 1,4-dithiothreitol (SIGMA-Aldrich) and protease/phosphatase inhibitor cocktail (Roche Diagnostics) on ice. For SDS-PAGE, 50 μg protein was loaded and blotted to a nitrocellulose membrane after separation. The following primary and secondary antibodies were used: ATPase Sarcoplasmic/Endoplasmic Reticulum Ca^2+^ Transporting 2 (ATP2A2/SERCA2, dilution 1:1000; Cell Signaling Technology #4388), CD80 (dilution 1:1000; Cell Signaling Technology #54521), Programmed Cell Death 1 (PD-1, dilution 1:1000; Cell Signaling Technology #84651), phospho-Phospholamban (Ser16/Thr17) (PLN, dilution 1:1000; Cell Signaling Technology #8496), PLN (dilution 1:1000; Cell Signaling Technology #14562), cardiac Troponin T (TnT, dilution 1:2000; proteintech 15513-1-AP), CD206 (dilution 1:200; R&D Systems AF2535) and donkey anti-rabbit IgG, peroxidase-linked species-specific whole antibody NA934V (dilution 1:3000; GE Healthcare). Chemiluminescence detection was carried out after incubation with enhanced chemiluminescence reagents (PerkinElmer) using the ChemiDoc™ MP system (Bio-Rad). Image LabV5.0 software (Bio-Rad) was used for quantification.

### Statistical analyses

Statistical analysis was performed using GraphPad Prism version 7.0 or 8.0 for Mac OS X (GraphPad Software, San Diego California United States). The Shapiro-Wilk test was used to test the data against the hypothesis of normal distribution. Continuous parametric data were expressed as mean ± SD, and in the case of non-parametric data, the median and interquartile range (IQR) were reported. Differences between groups were analysed by unpaired two-tailed Student’s t*-*test or Mann-Whitney *U* test dependent on data distribution. A *p*-value of <0.05 was considered statistically significant.

## Results

### PD-L1 inhibition showed no effect on cardiac function and morphology but was associated with increased survival of B16F10-GCV mice after tumor elimination and recovery

**FIGURE 2:**
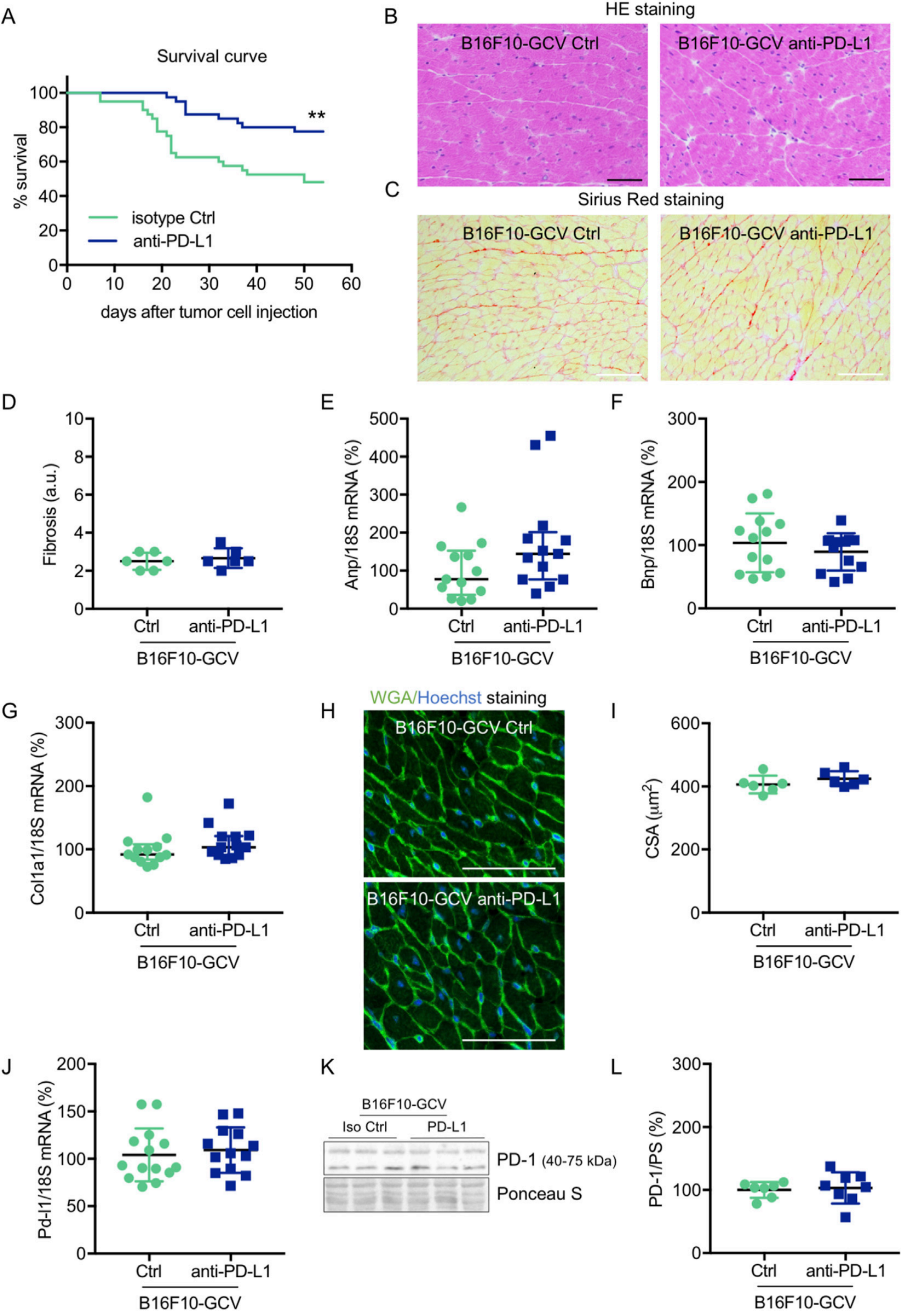
Cardiac phenotype of anti-PD-L1 treatment in B16F10-GCV mice after tumor eliminiation and recovery. **(A)** Kaplan-Meier survival curve for B16F10-GCV mice treated with anti-PD-L1 (N = 40) or isoptype Ctrl (Ctrl) (N = 40). Log-rank test was used for statistical survival analysis, **p < 0.01. **(B)** Representative sections with hematoxylin and eosin (HE) staining visualising cardiac morphology of LV cryosections from anti-PD-L1 or isotype Ctrl treated B16F10-GCV mice after tumor elimination and recovery; scale bars indicate 50 μm. **(C)** Representative images of Sirius red staining visualizing fibrosis and collagen deposits in LV cryosections from anti-PD-L1 or isotype Ctrl treated B16F10-GCV mice after tumor elimination and recovery; scale bars indicate 50 μm. **(D)** Quantification of fibrosis from anti-PD-L1 (N = 6) or isotype Ctrl (N = 6) treated B16F10-GCV LVs after tumor elimination and recovery in arbitrary units (a.u.). Dot plots summarizing **(E)** *Anp*, **(F)** *Bnp* and **(G)** *Col1a1* mRNA levels normalized to 18S RNA analysed by qRT-PCR in B16F10-GCV LVs treated with anti-PD-L1 (N = 13) or isotype Ctrl (N = 13). **(H)** Representative images of Fluorescein-labelled wheat germ agglutinin (WGA, green) and Hoechst 33528 (blue) staining of LV cryosections from anti-PD-L1 or isotype Ctrl treated B16F10-GCV mice after tumor elimination and recovery; scale bars indicate 50 μm. **(I)** Quantification of CSA from anti-PD-L1 (N = 6) or isotype Ctrl (N = 6) treated B16F10-GCV LVs after tumor elimination and recovery. **(J)** Dot plot summarizing *Pd-l1* mRNA levels normalized to 18S RNA analysed by qRT-PCR in B16F10-GCV LVs treated with anti-PD-L1 (N = 14) or isotype Ctrl (N = 13). **(K)** Representative cardiac PD-1 western blot and **(L)** dot plot summarizing quantification of cardiac PD-1 protein expression normalised to Ponceau S staining in cardiac tissue of B16F10-GCV LVs treated with anti-PD-L1 (N = 8) or isotype Ctrl (N = 7). Uncropped full length images are presented in SFig. 5A + B. **(D, F, G, I, J, L)** Gaussian distributed data were presented as mean ± SD and **(E)** not normally distributed data were presented as median and IQR, **p < 0.01 vs. B16F10-GCV isoptype Ctrl, unpaired two-tailed Student’s t*-*test or Mann-Whitney *U* test.

Male C57BL6/N mice were inoculated with the B16F10HSVtk-c melanoma cell line (B16F10 mice) as previously described (Pietzsch et al., 2021). Therefore, B16F10 mice were injected i.p. with 1 × 10^6^ cells to induce tumor growth (Figure 1A). At day 7 after tumor cell injection, mice (N = 80) were randomized to groups (N = 40) receiving anti-PD-L1 or isotype control (Ctrl) treatment. At day 14, GCV treatment was started until complete tumor eliminiation on day 34–36 (named as B16F10-GCV mice; Figure 1A). After 14 days of recovery (day 48–53), mice were subjected to echocardiography or PET-CT measurement followed by organ harvesting for further analysis (Figure 1A). Tumor progression and elimination was visualized by non-invasive BLI measurements, which demonstrated that PD-L1 targeting itself had no effect on tumor burden in surviving mice (Figures 1B, C). However, B16F10-GCV mice with anti-PD-L1 treatment showed an improved survival rate compared to isotype Ctrl treated mice 50 days after tumor inoculation and 43 days after starting anti-PDL1 or isotype Ctrl treatment (Figure 2A). In surviving mice 50 days after tumor inoculation, echocardiographic analyses demonstrated comparable cardiac function and LV dimensions in anti-PD-L1- and isotype Ctrl-treated B16F10-GCV mice (Table 1). In addition, LV tissue morphology, collagen content (as determined by Sirius Red staining), cardiac weights and cardiomyocyte cross-sectional area (CSA) revealed no alterations between both groups (Table 1, Figures 2B–D, G–I). Moreover, the cardiac mRNA expression of the hypertrophy and heart failure markers *Anp* and *Bnp* and the fibrosis marker *Col1a1* were not affected by PD-L1 inhibition (Figures 2D–F). Age-matched GCV control mice without cancer (GCV mice) were treated according to the same protocol with anti-PD-L1 or isotype Ctrl and showed the same cardiac function, dimensions, cardiac and body weights and gene expression as B16F10-GCV mice after tumor elimination and recovery (Supplementary Figures S1A–D, Table 1). Cardiac *Pd-l1* mRNA and PD-1 protein expression was not altered after tumor elimination and recovery in B16F10-GCV mice (Figures 2J–L; Supplementary Figures S5A, B).

**TABLE 1:** Cardiac function and dimensions, body weight and tibia length in B16F10-GCV and GCV mice treated anti-PD-L1 or isotype Ctrl after tumor elimination and recovery.

Parameters	GCV Isotype Ctrl (N = 12)	GCV Anti-PD-L1 (N = 9)	B16F10-GCV Isotype Ctrl (N = 9)	B16F10-GCV Anti-PD-L1 (N = 13)
FS (%)	35.1 ± 4.2	36.7 ± 3.7	32.2 ± 4.8	33.6 ± 5.7
LVEDD (mm)	4.2 ± 0.2	4.2 ± 0.2	4.3 ± 0.3	4.2 ± 0.3
LVESD (mm)	2.7 ± 0.2	2.7 ± 0.3	2.9 ± 0.3	2.8 ± 0.3
Heart rate (bpm), median (IQR)	606 (576–620)	595 (580–610)	602 (591–624)	590 (544–609)
Cardiac output (mL/min), median (IQR)	20.1 (17.3–21.2)	19.8 (18.5–22.2)	19.9 (17.2–21.6)	18.3 (17.2–20.6)
HW (mg)	110.5 ± 8.0	110.1 ± 5.9	108.8 ± 13.9	105.4 ± 11.3
BW (g)	26.8 ± 1.7	27.0 ± 1.6	26.3 ± 1.3	24.8 ± 2.1*
HW/BW ratio, median (IQR)	4.1 (4.1–4.2)	4.1 (3.9–4.2)	4.1 (3.9–4.2)	4.2 (4.2–4.4)
Tibia length (TL, mm), median (IQR)	16.8 (16.8–16.9)	16.8 (16.7–16.9)	16.8 (16.7–16.9)	16.8 (16.8–16.8)
HW/TL ratio, median (IQR)	6.5 (6.1–7.0)	6.6 (6.3–6.8)	6.4 (5.9–6.8)	6.1 (6.0–7.1)

Fractional shortening (FS), left ventricular end-diastolic diameter (LVEDD), left ventricular end-systolic diameter (LVESD), heart rate (beats per minute, bpm) determined in B-mode; cardiac output determined in M-mode from echocardiographic analyses and heart weight (HW), body weight (BW), HW/BW ratio, tibia length (TL) and HW/TL ratio determined in B16F10-GCV and GCV mice treated with anti-PD-L1 or isotype Ctrl (Ctrl) after tumor elimination and recovery. Data are shown as mean ± SD, or median (IQR), *p < 0.05 vs. GCV anti-PD-L1, using unpaired two-tailed Student’s t*-*test.

### Anti-PD-L1 treatment increased circulating TNNI3 levels and induced cardiac expression of αMHC and TnT after tumor elimination and recovery

**FIGURE 3:**
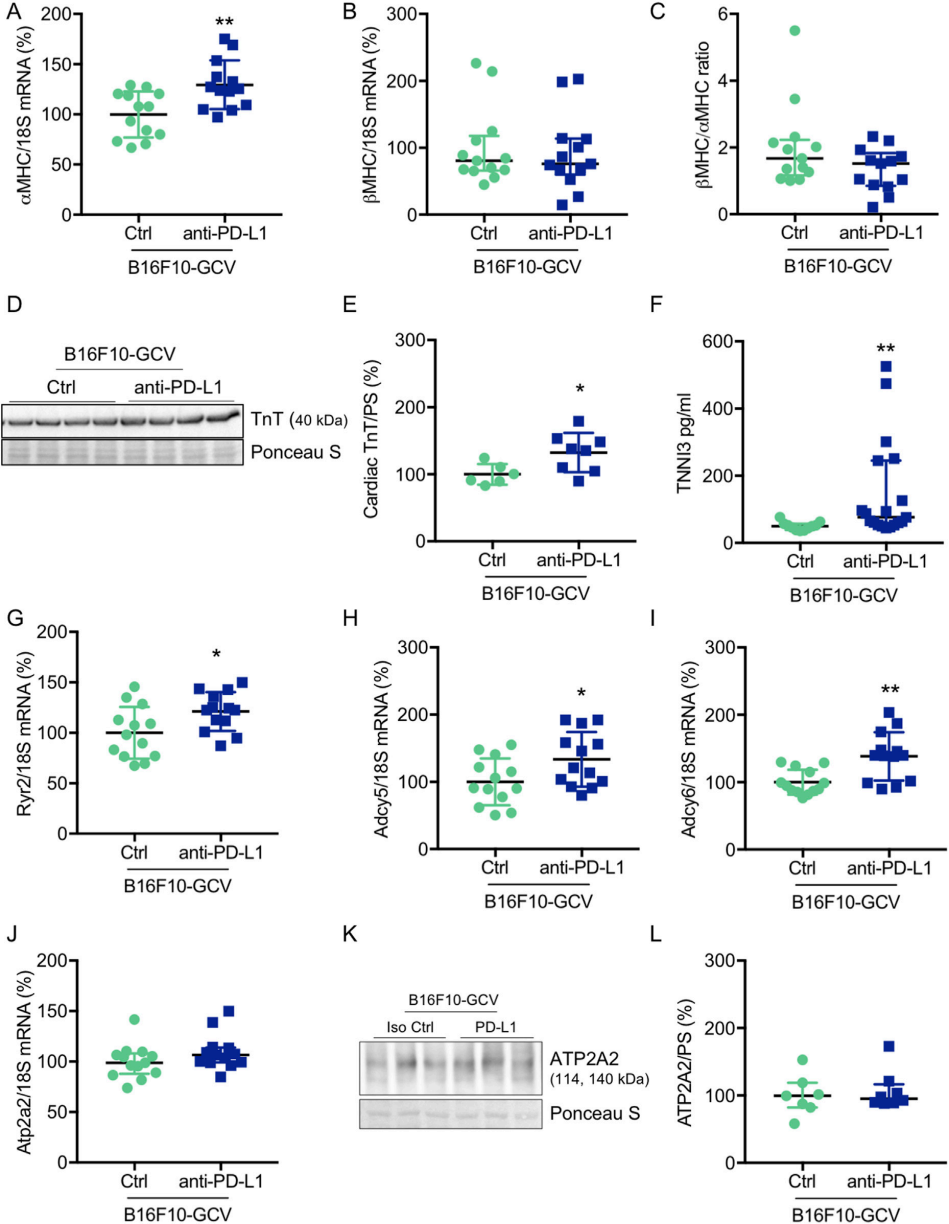
Cardiac effects of anti-PD-L1 treatment in B16F10-GCV mice. Dot plots summarizing **(A)** *αMHC* and **(B)** *βMHC* mRNA levels normalized to 18S RNA analysed by qRT-PCR in B16F10-GCV LVs treated with anti-PD-L1 (N = 13) or isotype Ctrl (N = 13). **(C)** Ratio of *βMHC*/*αMHC* mRNA expression of B16F10-GCV LVs treated with anti-PD-L1 (N = 13) or isotype Ctrl (N = 13). **(D)** Representative cardiac TnT western blot and **(E)** dot plot summarizing quantification of cardiac TnT protein expression normalised to Ponceau S staining in cardiac tissue of B16F10-GCV LVs treated with anti-PD-L1 (N = 8) or isotype Ctrl (N = 6). Uncropped full length images are presented in SFig. 6A + B. **(F)** The dot plots summarize circulating plasma troponin I type 3 (cardiac, TNNI3) levels from B16F10-GCV mice treated with anti-PD-L1 (N = 19) or isotype Ctrl (N = 11). Dot plots summarizing **(G)** *Atp2a2,* **(H)** *Adcy5,* **(I)** *Adcy6 and* **(J)** *Ryr2* mRNA levels normalized to 18S RNA analysed by qRT-PCR in B16F10-GCV LVs treated with anti-PD-L1 (N = 13) or isotype Ctrl (N = 13). **(K)** Representative cardiac ATP2A2 western blot and **(L)** dot plot summarizing quantification of cardiac ATP2A2 protein expression normalized to Ponceau S staining in cardiac tissue of B16F10-GCV LVs treated with anti-PD-L1 (N = 8) or isotype Ctrl (N = 7). Uncropped full length images are presented in SFig. 7A + B. **(A, E, H–J)** Gaussian distributed data were presented as mean ± SD and **(B, C, F, G, L)** not normally distributed data were presented as median and IQR, *p < 0.05,**p < 0.01 vs. B16F10-GCV isoptype Ctrl, unpaired two-tailed Student’s t*-*test or Mann-Whitney *U* test.

To investigate whether anti-PD-L1 treatment during tumor disease is inducing cardiac damage and remodeling processes after tumor elimination and recovery, cardiac transcripts and proteins in the LV and circulating plasma proteins of B16F10-GCV and GCV mice were measured. Indeed, anti-PD-L1 treatment in B16F10-GCV mice was associated with a significant increase in cardiac *αMHC* mRNA and TnT protein expression, and elevated plasma troponin I type 3 (cardiac, TNNI3) levels compared to isotype Ctrl treated B16F10-GCV mice, while *βMHC* mRNA expression or the ratio of *βMHC/αMHC* was not affected (Figures 3A–F; Supplementary Figures S6A, B). In addition, the cardiac mRNA expression of several myocardial genes involved in the contractile/calcium handling machinery including *ryanodine receptor 2* (*Ryr2*) and *adenylate cyclase* (*Adcy*) 5 and 6 was increased in anti-PD-L1-treated compared to isotype Ctrl-treated B16F10-GCV mice, while *ATPase sarcoplasmic/endoplasmic reticulum Ca* ^*2+*^ *transporting 2* (*Atp2a2*, also known as *Serca2*) mRNA expression was not affected. (Figures 3G–J). Moreover, cardiac ATP2A2 and phospholamban (PLN) protein expression or activation was not changed in anti-PD-L1-treated B16F10-GCV mice compared to isotype Ctrl-treated B16F10-GCV mice (Figures 3K, L; Supplementary Figures S2A–D, 5B, 7A, B, 8A–C). In contrast, anti-PD-L1 treatment in GCV mice without former cancer disease showed no alterations in the expression of these cardiac transcripts or proteins except for *Ryr2* mRNA expression (Supplementary Figures 1E-M, Supplementary Figures 6C, D). The *Ryr2* mRNA expression was also increased in the anti-PD-L1-treated GCV mice compared to isotype Ctrl GCV mice suggesting that anti-PD-L1 *per se* increases cardiac expression indepently of cancer disease.

In addition, to investigate putative myocardial oxidative stress, we determined the mRNA expression of *mangan superoxide dismutase* (*MnSOD*) levels and lipid peroxidation as a lipid marker for oxidative stress in LVs from B16F10-GCV mice treated with anti-PD-L1 or isotype Ctrl. No alterations were found in the cardiac *MnSOD* mRNA expression or in the detection of MDA as the end product of lipid peroxidation in B16F10-GCV LVs treated with anti-PD-L1 or isotype Ctrl (Supplementary Figures 2E, F).

### Anti-PD-L1 treatment increased cardiac mRNA expression of macrophage inflammatory markers and induced the production and secretion of cardiac cytokines and chemokines

**FIGURE 4:**
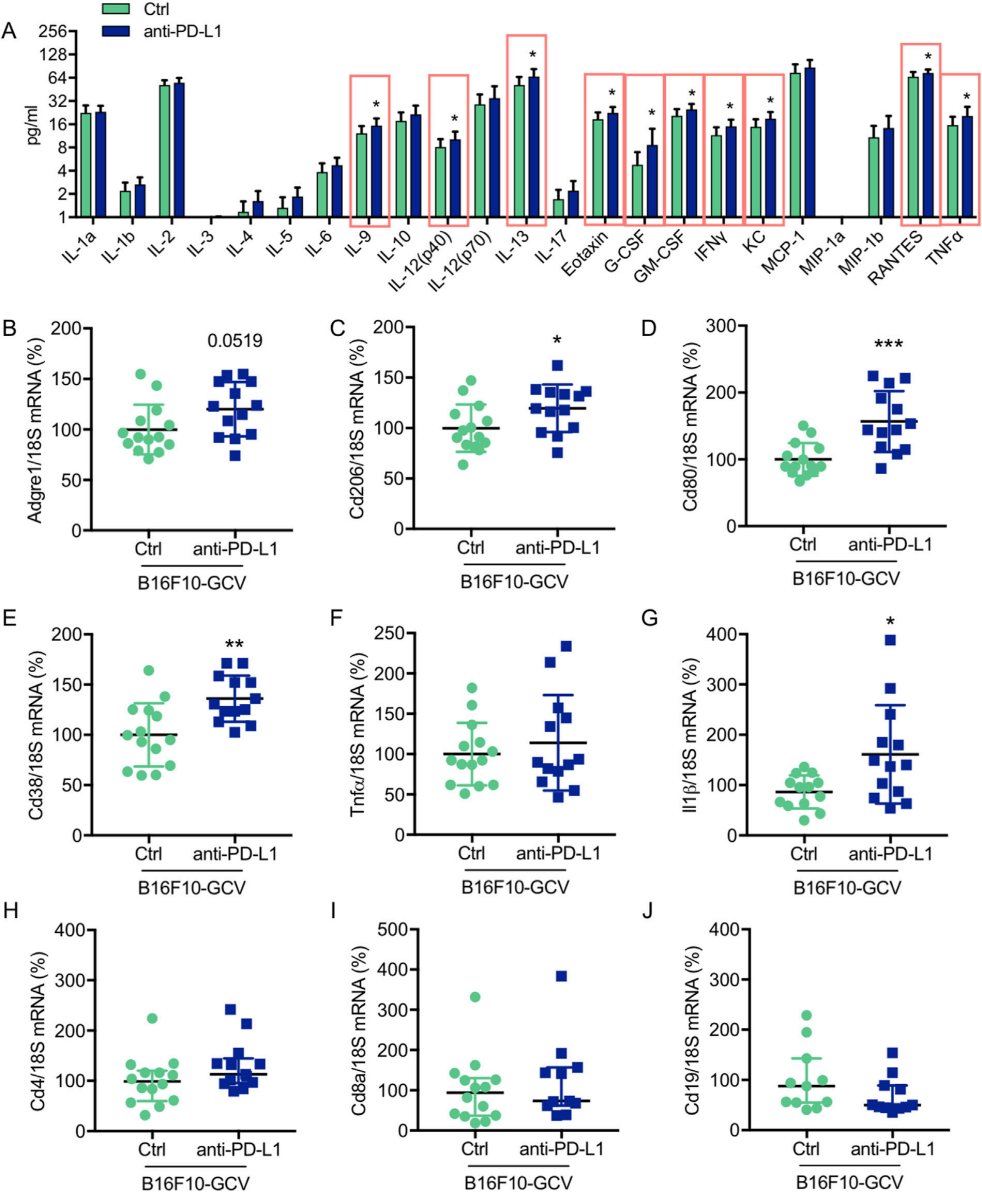
Anti-PD-L1 treatment promotes the secretion of inflammatory cytokines/chemokines and the expression of macrophage markers in LV tissue of B16F10-GCV mice **(A)** Induction of cytokine/chemokine protein concentration in LV tissue lysates of anti-PD-L1 treated B16F10-GCV mice (N = 13) compared to isotype Ctrl treated B16F10-GCV mice (N = 12), measured by Bio-Rad Multiplex Immunoassay System. Dot plots summarizing **(B)** *Adgre1*, **(C)** *Cd206,* **(D)** *Cd80,* **(E)** *Cd38*, **(F)** *Tnfα*, **(G)** *Il1β*, **(H)** *Cd4* and **(I)** *Cd8a* mRNA levels normalized to 18S RNA analysed by qRT-PCR in B16F10-GCV LVs treated with anti-PD-L1 (N = 13) or isotype Ctrl (N = 14). **(J)** Dot plot summarizing *Cd19* mRNA levels normalized to 18S RNA analysed by qRT-PCR in B16F10-GCV LVs treated with anti-PD-L1 (N = 11) or isotype Ctrl (N = 11). **(B–G)** Gaussian distributed data were presented as mean ± SD and **(H–J)** not normally distributed data were presented as median and IQR, *p < 0.05, **p < 0.01, ***p < 0.001 vs. B16F10-GCV isoptype Ctrl, unpaired two-tailed Student’s t*-*test or Mann-Whitney *U* test.

To investigate the effect of anti-PD-L1 treatment on inflammatory cyto- and chemokine production after melanoma cancer disease, a multiplex assay was performed to compare the concentration of 23 different inflammatory cytokines and chemokines in LV tissue of anti-PD-L1 and isotype Ctrl treated B16F10-GCV mice after tumor elimination and recovery (50 days after tumor induction and 43 days after starting anti-PD-L1 or isotype Ctrl treatment). Indeed, PD-L1 inhibition was associated with a significant induction of 11 cytokines/chemokines including interleukin (IL) 9, IL-12 (p40), IL-13, Eotaxin, granulocyte-colony stimulating factor (G-CSF), granulocyte-macrophage colony stimulating factor (GM-CSF), interferon γ (IFNγ), regulated on activation in normal T-Cell expressed and secreted (RANTES) and tumor necrosis factor α (TNFα) in LVs of B16F10-GCV mice (Figure 4A, Supplementary Figures 3A–V). In addition, mRNA expression of the macrophage markers *Cd206*, *Cd80* and *Cd38*, and the inflammatory marker *Il-1β* was increased in the LV of anti-PD-L1-treated compared to isotype Ctrl-treated B16F10-GCV mice (Figures 4B–J), while mRNA levels of the B cell marker *Cd19* and the T cell markers *Cd4* and *Cd8a* were not affected. Anti-PD-L1 treatment in GCV mice without former cancer disease revealed no alterations in the cardiac expression of inflammatory or macrophage markers, suggesting that the induction of the inflammatory response observed in B16F10-GCV mice treated with anti-PDL-1 is cancer dependent (Supplementary Figures S4A–I).

### Anti-PD-L1 treatment did not increase cardiac inflammation in B16F10-GCV mice after tumor elimination and recovery

**FIGURE 5:**
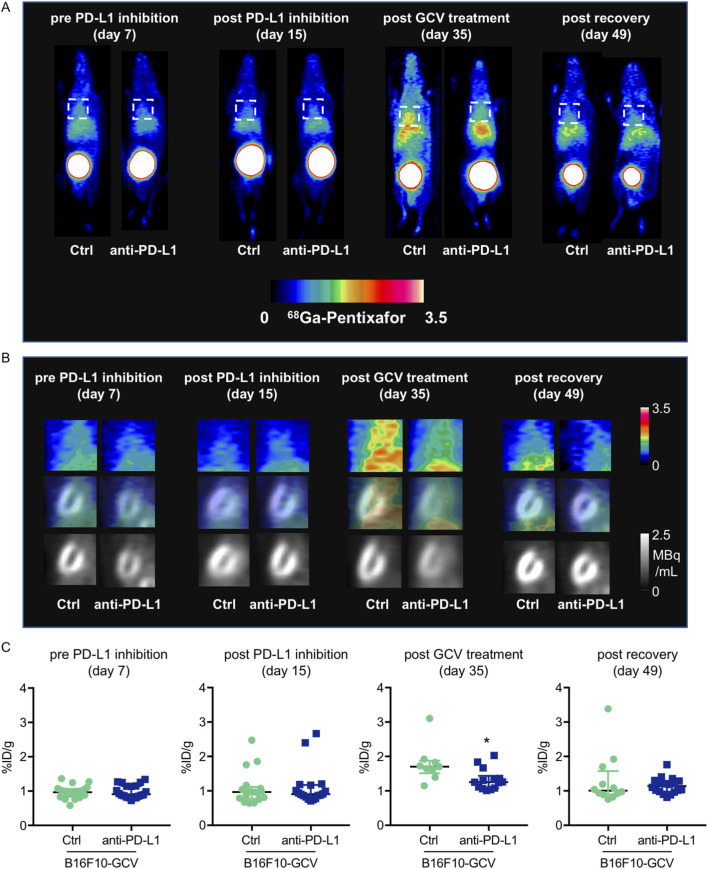
Measurements of inflammation by ^68^Ga-pentixafor in PET-CT of anti-PD-L1 or isotype Ctrl treated B16F10-GCV mice. **(A, B)** Inflammation was visualized before and after anti-PD-L1 treatment by ^68^Ga-pentixafor PET-CT and representative **(A)** whole body and **(B)** heart images are shown compared to isotype Ctrl B16F10-GCV mice. Regions of interest for data quantification are indicated by squares with dashed lines. **(C)** Quantitative analysis of percent injected dose per gram tissue in the heart (%ID/g) of ^68^Ga-pentixafor PET-CT, day 7 anti-PD-L1 (N = 20) vs. isotype Ctrl (N = 20), day 15 anti-PD-L1 (N = 18) vs. isotype Ctrl (N = 17), day 35 anti-PD-L1 (N = 14) vs. isotype Ctrl (N = 9) and day 49 anti-PD-L1 (N = 18) vs. isotype Ctrl (N = 12). Data were presented as median and IQR, statistical analysis was performed using Mann-Whitney *U* test, *p < 0.05 vs. B16F10-GCV isoptype Ctrl.

**FIGURE 6:**
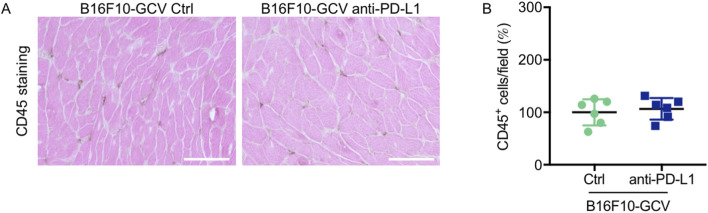
Inflammation by CD45 staining of anti-PD-L1 or isotype Ctrl treated B16F10-GCV mice. **(A)** Staining with the pan-inflammatory marker CD45 (brown, co-stained with eosin) showing inflammation in LV sections from anti-PD-L1-treated B16F10-GCV mice compared to isotype Ctrl-treated B16F10-GCV mice (50 days after tumor induction), scale bars indicate 50 μm. **(B)** Dot plot summarizing global inflammation (CD45^+^ cells) calculated as cells/field (in %) in LV sections from anti-PD-L1-treated B16F10-GCV mice (N = 6) compared to isotype Ctrl-treated B16F10-GCV mice (N = 6). Gaussian distributed data were presented as mean ± SD, n.s. vs. B16F10-GCV isoptype Ctrl, unpaired two-tailed Student’s t*-*test.

**FIGURE 7:**
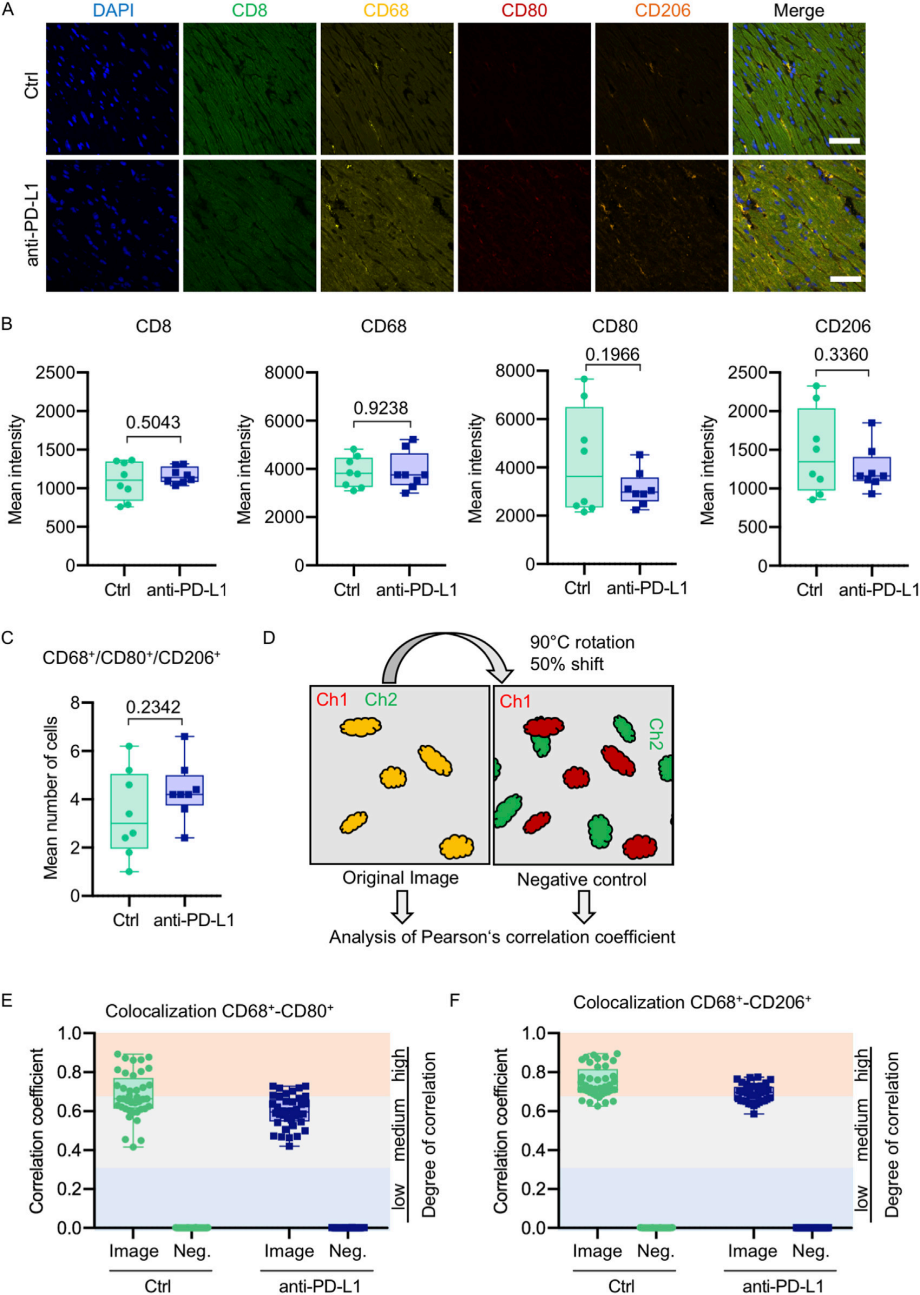
Anti-PD-L1 treatment was not associated with an increase of macrophages in the LV of B16F10-GCV mice. **(A)** Representative immunofluorescence images of immune cell markers in LV sections of anti-PD-L1 or isotype ctrl (Ctrl) treated B16F10-GCV mice. **(B)** Mean intensities of CD8, CD68, CD80 and CD206 fluorescence signal. One dot represents an average of five regions per animal (N = 8 animals per group). **(C)** Mean number of macrophages counted in five individual regions within a cardiac section. One dot represents the mean number of macrophages per area per animal (N = 8 animals per group). **(D)** Scheme of image processing. To generate a negative control image for supervision of random pixel colocalization the second channel (Ch) was rotated and shifted. Both the original image and the negative control image were then analyzed, respectively, for colocalization of fluorescent pixels in both channels. Colocalization analysis between **(E)** CD68 and CD80 or **(F)** CD68 and CD206 shown as Pearson’s correlation coefficient with respective negative control (Neg.). The colored background indicates the degree of colocalization. Each dot represents the Pearson’s correlation coefficient of one field of view in the heart section (n = 40, N = 8 animals per group). Box plots show median and interquartile range with whiskers from minimum to maximum values. Data were tested for Gaussian distribution and compared using unpaired two-tailed *t*-test.

To determine whether the observed increase in cardiac mRNA expression of inflammatory macrophage markers and cytokine/chemokine secretion in anti-PD-L1-treated B16F10-GCV mice is associated with an elevated number of inflammatory cells in the heart, the inflammatory phenotype/status was determined by serial PET-CT measurements for the CXCR4-ligand ^68^Ga-Pentixafor and fluorescence microsopy for T cell lymphocytes and macrophage makers (Figure 1A, Figures 5–7). PET-CT measurements of the CXCR4 ligand ^68^Ga-Pentixafor in B16F10-GCV mice were performed at the indicated timepoints (Figure 1A), including before and after PD-L1 inhibition, as well as after GCV treatment and following the recovery phase. Serial PET-CT measurements identified a temporal shift in ^68^Ga-pentixafor distribution over the progression from tumor inoculation to tumor elimination (Figures 1A, 5A). Diffuse CXCR4 signal in the LV was increased in both groups, anti-PD-L1 and isotype Ctrl treatment, after GCV-mediated tumor elimination, on day 35, but to a lesser extent in anti-PD-L1-treated animals (Figure 5B). This signal returned to baseline levels by 49 days after tumor induction. Quantitative analysis described no significant difference in the CXCR4 PET signal between PD-L1 inhibitor and isotype Ctrl after the treatment phase (day 15) or following the recovery phase (49 days after tumor induction, 42 days after starting anti-PD-L1 or isotype Ctrl treatment) (Figure 5C). By contrast, the increase of the ^68^Ga-Pentixafor signal after GCV administration (day 35) was moderately higher in isotype Ctrl-compared to anti-PD-L1-treated mice.

**FIGURE 8:**
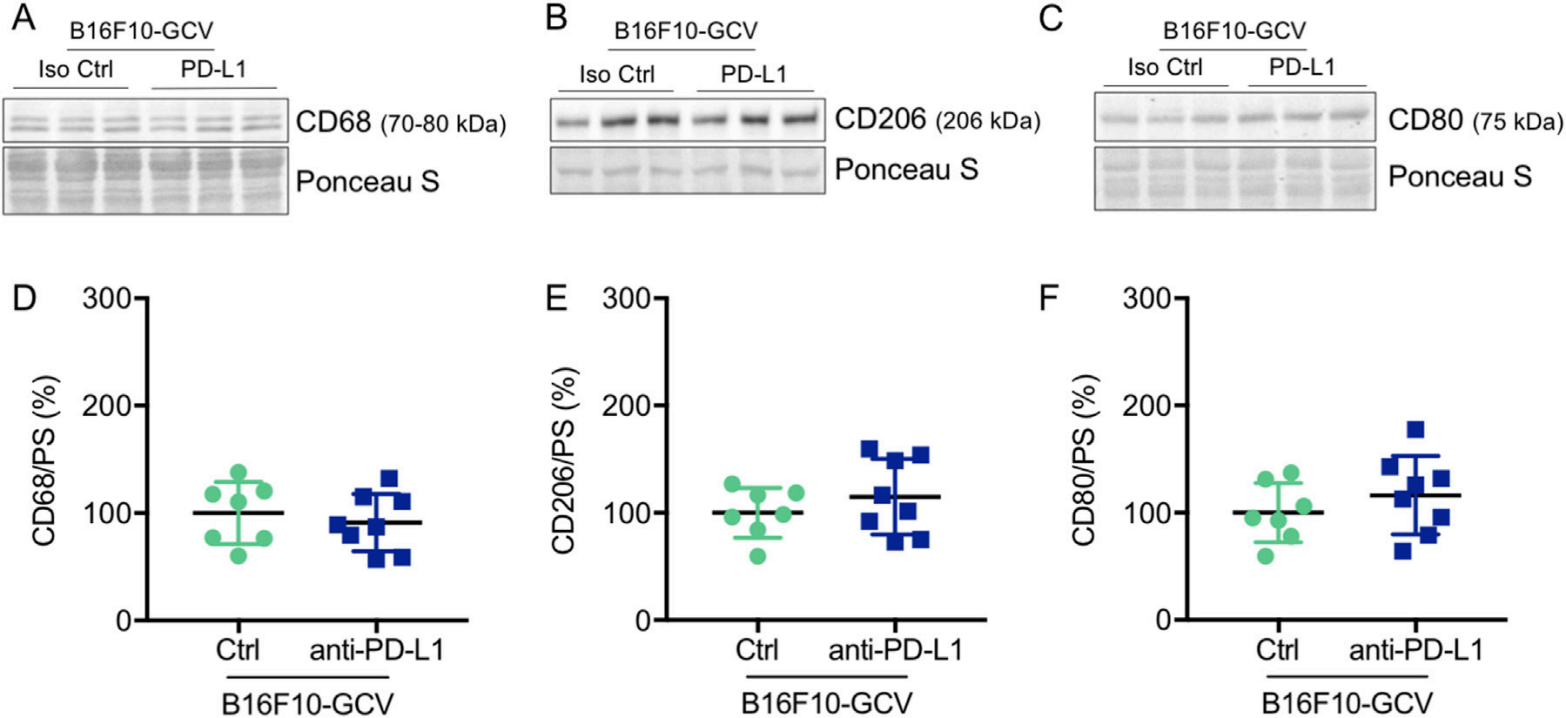
Anti-PD-L1 treatment was not associated with an increase in CD68, CD206 and CD80 protein expression in the LV of B16F10-GCV mice. Representative cardiac **(A)** CD68, **(B)** CD206 and **(C)** CD80 western blots and dot plots summarizing quantification of cardiac **(D)** CD68, **(E)** CD206 and **(F)** CD80 protein expression normalized to Ponceau S staining in cardiac tissue of B16F10-GCV LVs treated with anti-PD-L1 (N = 8) or isotype Ctrl (N = 7). Uncropped full length images are presented in SFig. 9A-E ans SFig. 8B. **(D–F)** Gaussian distributed data were presented as mean ± SD, n.s vs. B16F10-GCV isoptype Ctrl, unpaired two-tailed Student’s t*-*test.

Global inflammation determined by CD45 staining revealed a similar number of CD45^+^ cells in B16F10-GCV hearts treated with anti-PD-L1 or iso Ctrl at day 50 after tumor induction (43 days after starting anti-PD-L1 or isotype Ctrl treatment) (Figures 6A,B). Fluorescence immunostaining of cardiac sections at day 50 after tumor induction confirmed the lack of difference between both groups. Mean fluorescence intensities of CD8a, CD68, CD80 and CD206 were not significantly affected by anti-PD-L1 compared to isotype Ctrl treatment in B16F10-GCV mice (Figures 7A,B). In addition, comparable low numbers of macrophages were identified in the LV tissue of anti-PD-L1 and isotype Ctrl treated B16F10-GCV mice, as determined by CD68, CD80 and CD206 fluorescence (Figure 7C). Isotype Ctrl and anti-PD-L1-treated B16F10-GCV animals showed similar degrees of medium to high colocalization for both pro-and anti-inflammatory macrophage markers CD80 and CD206, respectively (Figures 7D–F). Moreover, cardiac CD68, CD206 and CD80 protein expression was not regulated in isotype Ctrl and anti-PD-L1-treated B16F10-GCV mice (Figures 8A–F; Supplementary Figures 8B, 9A–E).

## Discussion

In this study, we used a reversible melanoma mouse model (B16F10-GCV) to analyze potential ICI-mediated inflammatory processes in mice, which have been exposed to melanoma disease and are fully recovered without the use of cardiotoxic chemotherapy. In contrast to unchallenged mice, this model might be more representative for ICI administration in cancer patients. The suitability as a model for testing therapy-induced cardiac side effects has already been demonstrated for the known cardiotoxic agent doxorubicin (Pietzsch et al., 2021).

Several studies have suggested that the induction and pathogenesis of autoimmune myocarditis depends on environmental factors and immune-stimulated conditions (Moslehi et al., 2021; Nishimura et al., 2001; Tarrio et al., 2012; Gil-Cruz et al., 2019). For example, a combined knockout of PD-L1/PD-L2 in C57BL/6 mice, which present a rather autoimmune-resistant model, revealed no cardiac phenotype (Keir et al., 2006), whereas Grabie et al. have shown that a transient inflammation of the heart in C57BL/6 mice (Grabie et al., 2007), induced by transgenic cytotoxic T lymphocytes (CTLs) directed against cardiac myocytes, can be transformed to lethal myocarditis by PD-L1 blockade. Also, the deletion of PD-L1 or PD-1 in autoimmune-prone MRL-mice resulted in fatal myocarditis characterized by cardiac infiltration of CD8^+^ and CD4^+^ T cells and macrophages (Lucas et al., 2008; Wang et al., 2010).

Here, we analyzed the potential of PD-L1 inhibition for the development of ICI-induced cardiac inflammation in mice with melanoma cancer disease (B16F10-GCV mice). In our experiments, anti-PD-L1 treatment was associated with an increased survival of B16F10-mice during GCV-mediated tumor elimination, although BLI measurements revealed no difference in tumor burden of surviving anti-PD-L1-treated and control mice (Figures 1B, C). However, differences in size and distribution of tumors at the time of death in the deceased mice cannot be excluded and might have an impact on the survival rate. Indeed, the difference in survival is related to the time frame between 15 and 25 days after tumor induction, a periode after anti-PD-L1 or isotype Ctrl treatment and during the tumor elimination phase with GCV therapy. The effect of PD-L1 blockade on B16F10 tumor growth has been adressed by several reports with different outcomes. While Clark et al. (2016) and Ji et al. (2021) demonstrated a reduction of B16 tumors in NSG and C57BL/6 mice in response to PD-L1 inhibition, (Choi et al., 2018) and (Pilon-Thomas et al., 2010) showed no effect of anti-PD-L1 monotherapy on the size of B16F10 tumors in C57BL/6 mice. Thus, improved survival of anti-PD-L1-treated mice might represent a systemic effect of PD-L1 inhibition, rather than a direct effect on the tumor.

PD-L1 inhibition itself was not accompanied by cardiotoxic effects in terms of cardiac function and tissue morphology, confirming the observations of the above-mentioned studies, which have shown that blockade of the PD-1/PD-L1 axis itself is not sufficient to induce distinct myocarditis (Keir et al., 2006; Tarrio et al., 2012). In our former studies, we were able to demonstrate that the B16F10-GCV mouse model developed impaired cardiac function in the advanced cancer disease stage, which is reversible after tumor elimination and recovery (Pietzsch et al., 2021). Here, we could confirm the restored cardiac function of the B16F10-GCV model with anti-PD-L1 or isotype Ctrl treatment after recovery from tumor disease (50 days after tumor inoculation). In fact, we observed increased expression of *αMHC* and TnT, and elevated circulating TNNI3 levels in response to PD-L1 inhibition, but only in B16F10-GCV- and not in GCV-control mice. As mRNA expression of other markers for cardiac pathologic alterations, such as *βMHC*, and also *Anp*, *Bnp* and *Col1a1* in the LV tissue of B16F10-GCV mice was not affected by PD-L1 inhibition, this rather suggests beneficial remodeling in the heart of mice, which are recovered from tumor disease, than a pathologic effect of PD-L1 inhibition. It cannot be excluded that the combination of anti-PD-L1 treatment and melanoma cancer in the early stages of the disease induced cardiac damage, which was compensated after tumor elimination and recovery and did not result in functional cardiac impairment. Moreover, cardiac ATP2A2 and PLN protein expression and activation was not altered in anti-PD-L1-treated B16F10-GCV mice compared to isotype Ctrl-treated B16F10-GCV mice. The mRNA expression of *Adcy5* and *Adcy6*, which are crucial for β-adrenergic receptor (β-AR) signaling in the heart, is also specifically upregulated by anti-PD-L1 treatment in B16F10-GCV mice, but not in GCV-control mice. While ADCY5 has been reported to be involved in the regulation of oxidative stress (Lai et al., 2013), *Adcy5* upregulation in this study was not associated with transcriptional regulation of *MnSOD* or increased lipid peroxidation as a lipid marker for oxidative stress in B16F10-GCV mice. These data again emphasize that the cardiac phenotype described in this study is specific for PD-L1 inhibition in the context of melanoma disease and that cancer-free mouse models may not be sufficient to analyze ICI-mediated cardiotoxicity.

In contrast, the cardiac mRNA expression of *Ryr2* was increased in anti-PD-L1-treated B16F10-GCV and in GCV mice compared to isotype Ctrl demonstrating that anti PD-L1 *per se* may mediate the expression of several genes involved in the calcium handling independent of melanoma disease.

Numerous studies have demonstrated the induction of T cell-mediated autoimmune myocarditis by the release of cardiac myosin as a result of cardiomyocyte damage (Neu et al., 1987; Smith and Allen, 1991; Neumann et al., 1994; Li et al., 2004; Axelrod et al., 2022). In this study, induction of *αMHC* mRNA expression in the LV tissue of anti-PD-L1-treated mice was not accompanied by infiltration with T cells or macrophages, as determined by immunohistology.

However, we observed elevated plasma levels of cardiac TNNI3 in response to anti-PD-L1 treatment, indicating the damage of cardiomyocytes. Notably, this was only apparent in B16F10-GCV mice, while in GCV mice PD-L1 inhibition without tumor burden had no effect on *αMHC*, cardiac TnT and plasma TNNI3 levels. Furthermore, mRNA levels of the macrophage markers *Cd206*, *Cd38* and *Cd80*, as well as *Il-1β*, were increased in LV tissue of anti-PD-L1- compared to isotype Ctrl-treated animals, suggesting a mild inflammatory response to PD-L1 inhibition in the heart of B16F10-GCV mice. In GCV control mice, PD-L1 inhibition had no impact on mRNA levels of *Cd206*, *Cd38*, *Cd80* and *Il-1β*, highlighting the relevance of tumour biology and systemic impact of cancer on the side effects of PD-L1 inhibitors.

Multiplex analysis of cytokine production/secretion in the LV tissue of B16F10-GCV mice confirmed an inflammatory response upon PD-L1 inhibition, which is shaped by cytokines/chemokines that are secreted by several types of immune cells, including macrophages, and are crucial for the promotion of T cell responses. Although an induction of a mild inflammatory response has been demonstrated in anti-PD-L1-treated mice, no accumulation of inflammatory cells was detected in the LV tissue of anti-PD-L1-treated compared to isotype-treated control animals by serial PET-CT/^68^Ga-Pentixafor measurements or immunohistochemistry. This observation was confirmed by immunohistology data, which did not hint at infiltrating macrophages or T cells in the LV tissue of anti-PD-L1-treated animals, and by Western Blot analysis, which showed comparable protein expression of CD68, CD80 and CD206 in the LV tissue of the animals from both groups. As the cytokine expression profile that was determined for PD-L1 inhibition is dominated by cytokines and chemokines of an early immune response, with IL-1 and TNFα belonging to the first cytokines released as part of the innate immune response (Bazzoni and Beutler, 1996; Dinarello, 1997), we cannot exclude a later T-cell reaction. PET-CT imaging was performed at time points, covering a period of time (8–42 days following the first ICI application) that represents the median time of onset for ICI-induced myocarditis in clinical studies (30–34 days after starting ICI) (Mahmood et al., 2018; Salem et al., 2018).

Notably, the reported fatality rates were much higher when anti-PD-L1 treatment was combined with anti-CTLA4 treatment compared to anti-PD-L1 monotherapy (Moslehi et al., 2018). Also germline genetic variation of ICI receiving patients is discussed as a potential determinant for occurrence of ICI-induced IRAE (Chin et al., 2022). Therefore, it might be possible that another mouse strain might be more suitable for the display of cardiotoxic effects in this experimental setting.

Surprisingly, in both treatment groups, PET-CT measurements revealed an accumulation of ^68^Ga-Pentixafor-labeled inflammatory cells following GCV-mediated tumor elimination, which was almost reduced to baseline in recovered animals. This effect was much more pronounced in isotype Ctrl- compared to anti-PD-L1-treated animals and possibly reflects an inflammatory response induced by the tumor or massive GCV-induced lysis of tumor cells (Balkwill and Mantovani, 2001; Mantovani et al., 2008; Vakkila and Lotze, 2004; Lupusoru et al., 2022). Inhibition of PD-L1 might be protective in this context, which is also supported by the reduced mortality of anti-PD-L1-treated B16F10-GCV mice during the tumor elimination phase.

## Conclusion

The use of antibody therapies directed against PD-L1 during melanoma cancer disease may mediate longlasting molecular changes in the heart, such as the generation of circulating TNNI3 as a result of moderate cardiac damage and the induction of inflammatory macrophage genes. These alterations did not affect cardiac functions of the animals in our study after tumor elimination and recovery, but may make the heart more susceptible to additional stress over the course of life and thus may partly contribute to late cardiotoxicity. Without a second hit, like an infection that triggers the inflammatory response, anti-PD-L1 treatment appears to be tolerated in the heart in the preclinical melanoma mouse model. Moreover, PD-L1 inhibition in the absence of tumor is unlikely to reflect the systemic immune and physiological response that occurs in combination with cancer, ICI therapy and tumor eliminiation. Thus, the reversible melanoma mouse model B16F10-GCV provides a useful tool to study cardiac side effects of anti-cancer treatments.

## Data Availability

The raw data supporting the conclusions of this article will be made available by the authors, without undue reservation.

## References

[B1] Axelrod M. L. Meijers W. C. Screever E. M. Qin J. Carroll M. G. Sun X. (2022). T cells specific for α-myosin drive immunotherapy-related myocarditis. Nature 611, 818–826. 10.1038/s41586-022-05432-3 36385524 PMC9930174

[B2] Balkwill F. Mantovani A. (2001). Inflammation and cancer: back to Virchow? Lancet 357, 539–545. 10.1016/S0140-6736(00)04046-0 11229684

[B3] Bazzoni F. Beutler B. (1996). The tumor necrosis factor ligand and receptor families. N. Engl. J. Med. 334, 1717–1725. 10.1056/NEJM199606273342607 8637518

[B4] Chin I. S. Khan A. Olsson-Brown A. Papa S. Middleton G. Palles C. (2022). Germline genetic variation and predicting immune checkpoint inhibitor induced toxicity. NPJ Genom Med. 7, 73. 10.1038/s41525-022-00345-6 36564402 PMC9789157

[B5] Choi J. Beaino W. Fecek R. J. Fabian K. P. L. Laymon C. M. Kurland B. F. (2018). Combined VLA-4-targeted radionuclide therapy and immunotherapy in a mouse model of melanoma. J. Nucl. Med. 59, 1843–1849. 10.2967/jnumed.118.209510 29959213 PMC6278902

[B6] Clark C. A. Gupta H. B. Sareddy G. Pandeswara S. Lao S. Yuan B. (2016). Tumor-intrinsic PD-L1 signals regulate cell growth, pathogenesis, and autophagy in ovarian cancer and melanoma. Cancer Res. 76, 6964–6974. 10.1158/0008-5472.CAN-16-0258 27671674 PMC5228566

[B7] De Mello R. A. Veloso A. F. Esrom Catarina P. Nadine S. Antoniou G. (2017). Potential role of immunotherapy in advanced non-small-cell lung cancer. Onco Targets Ther. 10, 21–30. 10.2147/OTT.S90459 28031719 PMC5179204

[B8] Dinarello C. A. (1997). Interleukin-1. Cytokine Growth Factor Rev. 8, 253–265. 10.1016/s1359-6101(97)00023-3 9620641

[B9] Gil-Cruz C. Perez-Shibayama C. De Martin A. Ronchi F. Van Der Borght K. Niederer R. (2019). Microbiota-derived peptide mimics drive lethal inflammatory cardiomyopathy. Science 366, 881–886. 10.1126/science.aav3487 31727837

[B10] Glasenapp A. Derlin K. Gutberlet M. Hess A. Ross T. L. Wester H. J. (2021). Molecular imaging of inflammation and fibrosis in pressure overload heart failure. Circ. Res. 129, 369–382. 10.1161/CIRCRESAHA.120.318539 34074134

[B11] Grabie N. Gotsman I. Dacosta R. Pang H. Stavrakis G. Butte M. J. (2007). Endothelial programmed death-1 ligand 1 (PD-L1) regulates CD8+ T-cell mediated injury in the heart. Circulation 116, 2062–2071. 10.1161/CIRCULATIONAHA.107.709360 17938288

[B12] Heimerl M. Sieve I. Ricke-Hoch M. Erschow S. Battmer K. Scherr M. (2020). Neuraminidase-1 promotes heart failure after ischemia/reperfusion injury by affecting cardiomyocytes and invading monocytes/macrophages. Basic Res. Cardiol. 115, 62. 10.1007/s00395-020-00821-z 32975669 PMC7519006

[B13] Hilfiker-Kleiner D. Hilfiker A. Fuchs M. Kaminski K. Schaefer A. Schieffer B. (2004). Signal transducer and activator of transcription 3 is required for myocardial capillary growth, control of interstitial matrix deposition, and heart protection from ischemic injury. Circ. Res. 95, 187–195. 10.1161/01.RES.0000134921.50377.61 15192020

[B14] Hoch M. Fischer P. Stapel B. Missol-Kolka E. Sekkali B. Scherr M. (2011). Erythropoietin preserves the endothelial differentiation capacity of cardiac progenitor cells and reduces heart failure during anticancer therapies. Cell. Stem Cell. 9, 131–143. 10.1016/j.stem.2011.07.001 21816364

[B15] Ji S. Lee J. Lee E. S. Kim D. H. Sin J. I. (2021). B16 melanoma control by anti-PD-L1 requires CD8+ T cells and NK cells: application of anti-PD-L1 Abs and Trp2 peptide vaccines. Hum. Vaccin Immunother. 17, 1910–1922. 10.1080/21645515.2020.1866951 33522416 PMC8189047

[B16] Keir M. E. Liang S. C. Guleria I. Latchman Y. E. Qipo A. Albacker L. A. (2006). Tissue expression of PD-L1 mediates peripheral T cell tolerance. J. Exp. Med. 203, 883–895. 10.1084/jem.20051776 16606670 PMC2118286

[B17] Lai L. Yan L. Gao S. Hu C. L. Ge H. Davidow A. (2013). Type 5 adenylyl cyclase increases oxidative stress by transcriptional regulation of manganese superoxide dismutase via the SIRT1/FoxO3a pathway. Circulation 127, 1692–1701. 10.1161/CIRCULATIONAHA.112.001212 23536361 PMC3980473

[B18] Li Y. Heuser J. S. Kosanke S. D. Hemric M. Cunningham M. W. (2004). Cryptic epitope identified in rat and human cardiac myosin S2 region induces myocarditis in the Lewis rat. J. Immunol. 172, 3225–3234. 10.4049/jimmunol.172.5.3225 14978130

[B19] Lucas J. A. Menke J. Rabacal W. A. Schoen F. J. Sharpe A. H. Kelley V. R. (2008). Programmed death ligand 1 regulates a critical checkpoint for autoimmune myocarditis and pneumonitis in MRL mice. J. Immunol. 181, 2513–2521. 10.4049/jimmunol.181.4.2513 18684942 PMC2587295

[B20] Lupusoru G. Ailincai I. Fratila G. Ungureanu O. Andronesi A. Lupusoru M. (2022). Tumor lysis syndrome: an endless challenge in onco-nephrology. Biomedicines 10, 1012. 10.3390/biomedicines10051012 35625753 PMC9138780

[B21] Mahmood S. S. Fradley M. G. Cohen J. V. Nohria A. Reynolds K. L. Heinzerling L. M. (2018). Myocarditis in patients treated with immune checkpoint inhibitors. J. Am. Coll. Cardiol. 71, 1755–1764. 10.1016/j.jacc.2018.02.037 29567210 PMC6196725

[B22] Mahoney K. M. Freeman G. J. Mcdermott D. F. (2015). The next immune-checkpoint inhibitors: PD-1/PD-L1 blockade in melanoma. Clin. Ther. 37, 764–782. 10.1016/j.clinthera.2015.02.018 25823918 PMC4497957

[B23] Mantovani A. Allavena P. Sica A. Balkwill F. (2008). Cancer-related inflammation. Nature 454, 436–444. 10.1038/nature07205 18650914

[B24] Morton D. B. Griffiths P. H. (1985). Guidelines on the recognition of pain, distress and discomfort in experimental animals and an hypothesis for assessment. Vet. Rec. 116, 431–436. 10.1136/vr.116.16.431 3923690

[B25] Moslehi J. Lichtman A. H. Sharpe A. H. Galluzzi L. Kitsis R. N. (2021). Immune checkpoint inhibitor-associated myocarditis: manifestations and mechanisms. J. Clin. Investig. 131, e145186. 10.1172/JCI145186 33645548 PMC7919710

[B26] Moslehi J. J. Salem J. E. Sosman J. A. Lebrun-Vignes B. Johnson D. B. (2018). Increased reporting of fatal immune checkpoint inhibitor-associated myocarditis. Lancet 391, 933. 10.1016/S0140-6736(18)30533-6 PMC6668330 29536852

[B27] Neilan T. G. Rothenberg M. L. Amiri-Kordestani L. Sullivan R. J. Steingart R. M. Gregory W. (2018). Myocarditis associated with immune checkpoint inhibitors: an expert consensus on data gaps and a call to action. Oncologist 23, 874–878. 10.1634/theoncologist.2018-0157 29802220 PMC6156187

[B28] Neu N. Rose N. R. Beisel K. W. Herskowitz A. Gurri-Glass G. Craig S. W. (1987). Cardiac myosin induces myocarditis in genetically predisposed mice. J. Immunol. 139, 3630–3636. 10.4049/jimmunol.139.11.3630 3680946

[B29] Neumann D. A. Rose N. R. Ansari A. A. Herskowitz A. (1994). Induction of multiple heart autoantibodies in mice with coxsackievirus B3- and cardiac myosin-induced autoimmune myocarditis. J. Immunol. 152, 343–350. 10.4049/jimmunol.152.1.343 8254202

[B30] Nishimura H. Okazaki T. Tanaka Y. Nakatani K. Hara M. Matsumori A. (2001). Autoimmune dilated cardiomyopathy in PD-1 receptor-deficient mice. Science 291, 319–322. 10.1126/science.291.5502.319 11209085

[B31] Palaskas N. Lopez-Mattei J. Durand J. B. Iliescu C. Deswal A. (2020). Immune checkpoint inhibitor myocarditis: pathophysiological characteristics, diagnosis, and treatment. J. Am. Heart Assoc. 9, e013757. 10.1161/JAHA.119.013757 31960755 PMC7033840

[B32] Pietzsch S. Wohlan K. Thackeray J. T. Heimerl M. Schuchardt S. Scherr M. (2021). Anthracycline-free tumor elimination in mice leads to functional and molecular cardiac recovery from cancer-induced alterations in contrast to long-lasting doxorubicin treatment effects. Basic Res. Cardiol. 116, 61. 10.1007/s00395-021-00902-7 34669013 PMC8528750

[B33] Pilon-Thomas S. Mackay A. Vohra N. Mule J. J. (2010). Blockade of programmed death ligand 1 enhances the therapeutic efficacy of combination immunotherapy against melanoma. J. Immunol. 184, 3442–3449. 10.4049/jimmunol.0904114 20194714 PMC2913584

[B34] Pincha M. Salguero G. Wedekind D. Sundarasetty B. S. Lin A. Kasahara N. (2011). Lentiviral vectors for induction of self-differentiation and conditional ablation of dendritic cells. Gene Ther. 18, 750–764. 10.1038/gt.2011.15 21412283 PMC3155152

[B35] Salem J. E. Manouchehri A. Moey M. Lebrun-Vignes B. Bastarache L. Pariente A. (2018). Cardiovascular toxicities associated with immune checkpoint inhibitors: an observational, retrospective, pharmacovigilance study. Lancet Oncol. 19, 1579–1589. 10.1016/S1470-2045(18)30608-9 30442497 PMC6287923

[B36] Salloum F. N. Tocchetti C. G. Ameri P. Ardehali H. Asnani A. De Boer R. A. (2023). Priorities in cardio-oncology basic and translational science: GCOS 2023 symposium proceedings: JACC: CardioOncology state-of-the-art review. JACC CardioOncol 5, 715–731. 10.1016/j.jaccao.2023.08.003 38205010 PMC10774781

[B37] Schardt J. (2020). The use of immune checkpoint inhibitors in routine oncology. Z Rheumatol. 79, 809–817. 10.1007/s00393-020-00876-2 32936368 PMC7653782

[B38] Smith S. C. Allen P. M. (1991). Myosin-induced acute myocarditis is a T cell-mediated disease. J. Immunol. 147, 2141–2147. 10.4049/jimmunol.147.7.2141 1918949

[B39] Tarrio M. L. Grabie N. Bu D. X. Sharpe A. H. Lichtman A. H. (2012). PD-1 protects against inflammation and myocyte damage in T cell-mediated myocarditis. J. Immunol. 188, 4876–4884. 10.4049/jimmunol.1200389 22491251 PMC3345066

[B40] Thackeray J. T. Derlin T. Haghikia A. Napp L. C. Wang Y. Ross T. L. (2015). Molecular imaging of the chemokine receptor CXCR4 after acute myocardial infarction. JACC Cardiovasc Imaging 8, 1417–1426. 10.1016/j.jcmg.2015.09.008 26577262

[B41] Thackeray J. T. Pietzsch S. Stapel B. Ricke-Hoch M. Lee C. W. Bankstahl J. P. (2017). Insulin supplementation attenuates cancer-induced cardiomyopathy and slows tumor disease progression. JCI Insight 2, e93098. 10.1172/jci.insight.93098 28515362 PMC5436547

[B42] Tomicic M. T. Thust R. Kaina B. (2002). Ganciclovir-induced apoptosis in HSV-1 thymidine kinase expressing cells: critical role of DNA breaks, Bcl-2 decline and caspase-9 activation. Oncogene 21, 2141–2153. 10.1038/sj.onc.1205280 11948397

[B43] Vakkila J. Lotze M. T. (2004). Inflammation and necrosis promote tumour growth. Nat. Rev. Immunol. 4, 641–648. 10.1038/nri1415 15286730

[B44] Varricchi G. Galdiero M. R. Marone G. Criscuolo G. Triassi M. Bonaduce D. (2017). Cardiotoxicity of immune checkpoint inhibitors. ESMO Open 2, e000247. 10.1136/esmoopen-2017-000247 29104763 PMC5663252

[B45] Wang J. Okazaki I. M. Yoshida T. Chikuma S. Kato Y. Nakaki F. (2010). PD-1 deficiency results in the development of fatal myocarditis in MRL mice. Int. Immunol. 22, 443–452. 10.1093/intimm/dxq026 20410257

