# Supplementary material for: Immune-checkpoint-inhibitor therapy directed against PD-L1 is tolerated in the heart without manifestation of cardiac inflammation in a preclinical reversible melanoma mouse model
Source: Front Mol Med. 2025 Jan 6;4:1487526. doi: 10.3389/fmmed.2024.1487526 (PMC11743445; doi:10.3389/fmmed.2024.1487526)
Supplement: Supplementary file 1 [file Presentation1.pdf]

*Supplementary Material*

# Immune-checkpoint-inhibitor therapy directed against PD-L1 is tolerated in the heart without manifestation of cardiac inflammation in a preclinical reversible melanoma mouse model

Caroline Schoenherr[1,2*], Stefan Pietzsch[1,3*], Cristina Barca[4], Franziska E. Müller[5], Frauke S. Bahr[5], Martina Kasten[1], Andre Zeug[5], Sergej Erschow[1], Christine S. Falk[6], Evgeni Ponimaskin[5], James T. Thackeray[4], Denise Hilfiker-Kleiner[1,7], and Melanie Ricke-Hoch[1#]

[1]Department of Cardiology and Angiology, Hannover Medical School, Hannover, Germany
[2]Department of Hematology, Hemostasis, Oncology and Stem Cell Transplantation, Hannover Medical School, Hannover, Germany
[3]Department of Human Genetics, Hannover Medical School, Hannover, Germany
[4]Department of Nuclear Medicine, Hannover Medical School, Hannover, Germany
[5]Department of Cellular Neurophysiology, Hannover Medical School, Hannover, Germany
[6]Institute of Transplant Immunology, IFB-Tx, Hannover Medical School, Hannover, Germany
[7]Department of Cardiovascular Complications of Oncologic Therapies, Medical Faculty of the Philipps University Marburg, Marburg, Germany

[*]Equally contributing first authors

**[#]Corresponding author:** Melanie Ricke-Hoch
Department of Cardiology and Angiology,
Hannover Medical School
Carl-Neuberg Str. 1
30625 Hannover, Germany
Phone: +49-511-532-2531
E-mail: Hoch.melanie@mh-hannover.de

# Supplementary figures

## Supplementary Figure 1

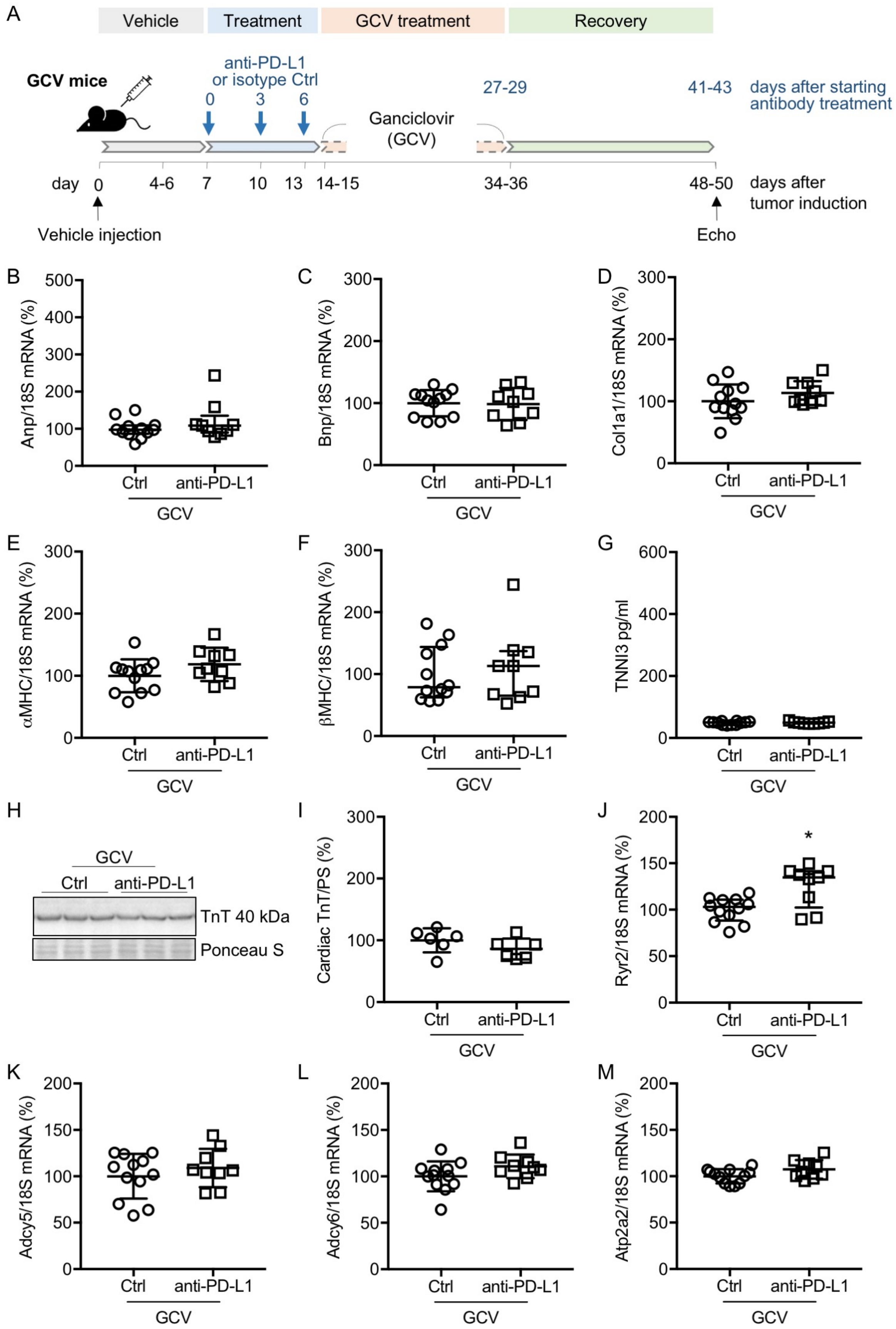

**Supplementary Figure 1** Cardiac effects of anti-PD-L1 treatment in GCV mice. (A) Scheme for anti-PD-L1 or isotype Ctrl treatment in GCV mice. Dot plots summarizing (B) *Anp*, (C) *Bnp*, (D) *Col1a1*, (E) *αMHC* and (F) *βMHC* mRNA levels normalised to 18S RNA analysed by qRT-PCR in GCV LVs treated with anti-PD-L1 (N=9) or isotype Ctrl (N=12). (G) The dot plots summarize circulating plasma troponin I type 3 (cardiac, TNNI3) levels from GCV mice treated with anti-PD-L1 (N=9) or isotype Ctrl (N=12). (H) Representative cardiac TnT Western blot and (I) dot plot summarising quantification of cardiac TnT protein expression normalised to Ponceau S staining in cardiac tissue of GCV LVs treated with anti-PD-L1 (N=6) or isotype Ctrl (N=6). Uncropped full length images are presented in SFig. 6C+D. Dot plots summarizing (J) *Ryr2*, (K) *Adcy5*, (L) *Adcy6* and (M) *Atp2a2* mRNA levels normalised to 18S RNA analysed by qRT-PCR in GCV LVs treated with anti-PD-L1 (N=9) or isotype Ctrl (N=12). (C-E, G, I, K-M) Gaussian distributed data were presented as mean±SD and (B, F, J) not normally distributed data were presented as median and IQR, *P<0.05 vs GCV isoptype Ctrl, unpaired two-tailed Student's *t* test or Mann-Whitney *U* test.

**Supplementary Figure 2**

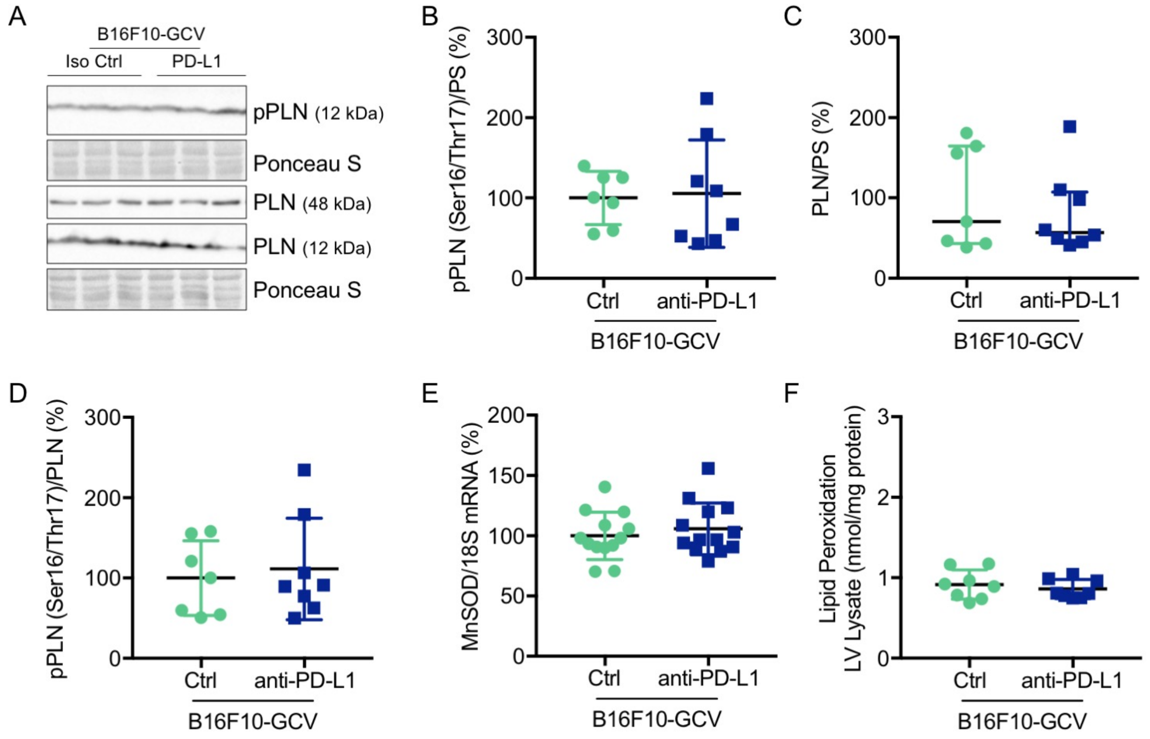


**Supplementary Figure 2** Cardiac PLN protein expression after anti-PD-L1 treatment in B16F10-GCV mice. (A) Representative cardiac pPLN, PLN and Ponceau S western blots and dot plot summarising quantification of cardiac (B) pPLN and (C) PLN protein expression normalised to Ponceau S staining, and (D) pPLN/PLN protein expression in cardiac tissue of B16F10-GCV LVs treated with anti-PD-L1 (N=8) or isotype Ctrl (N=7). Uncropped full length images are presented in SFig. 8A-C and 5B. (E) Dot plots summarizing *MnSOD* mRNA levels normalised to 18S RNA analysed by qRT-PCR in B16F10-GCV LVs treated with anti-PD-L1 (N=13) or isotype Ctrl (N=13). (F) Dot plots summarizing lipid peroxidation (nmol/mg protein) as a lipid marker of oxidative stress in B16F10-GCV LVs treated with anti-PD-L1 (N=8) or isotype Ctrl (N=8). (B, D-F) Gaussian distributed data were presented as mean±SD and (C) not normally distributed data were presented as median and IQR, not significant (n.s.), unpaired two-tailed Student's *t* test or Mann-Whitney *U* test.

**Supplementary Figure 3**

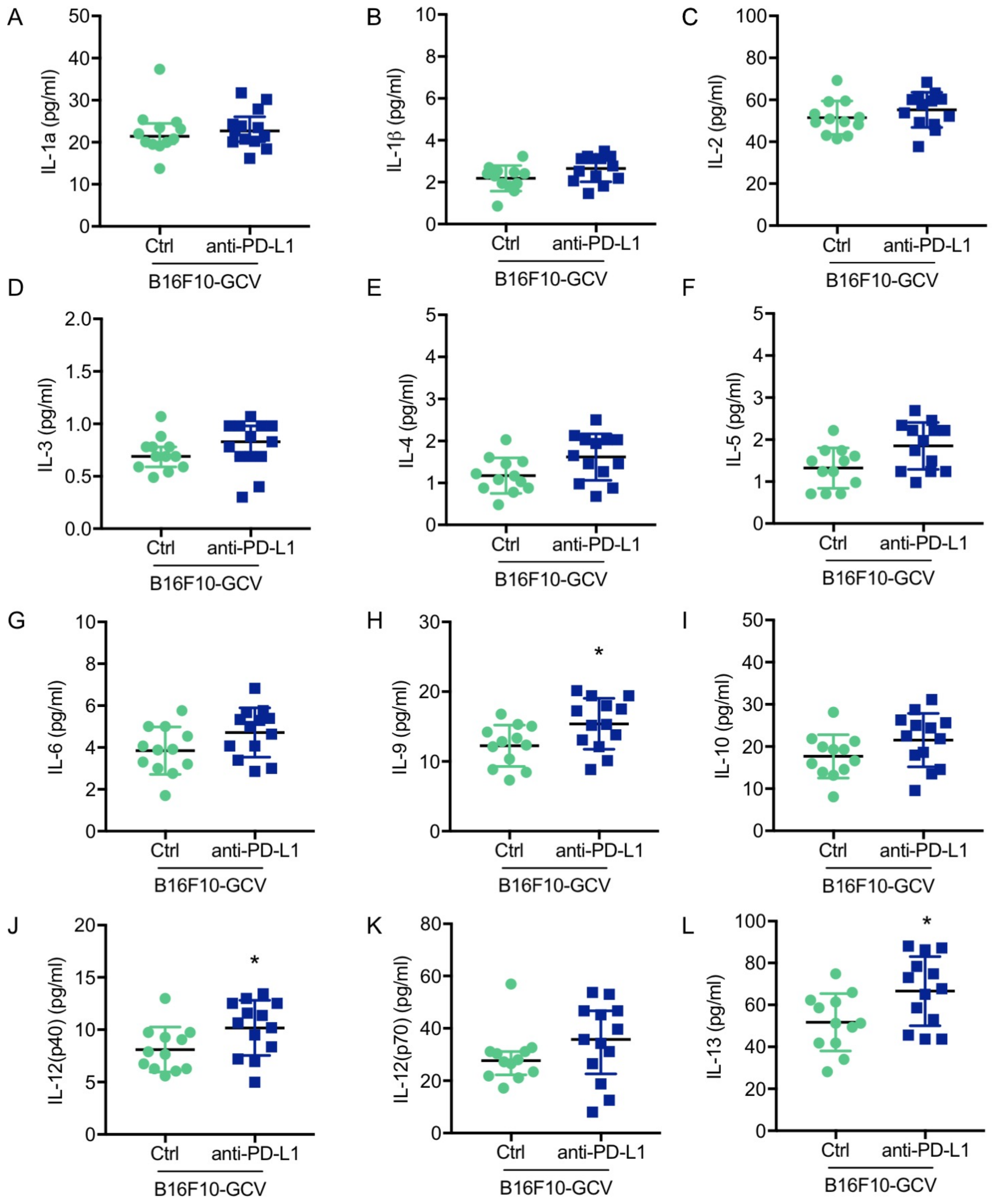

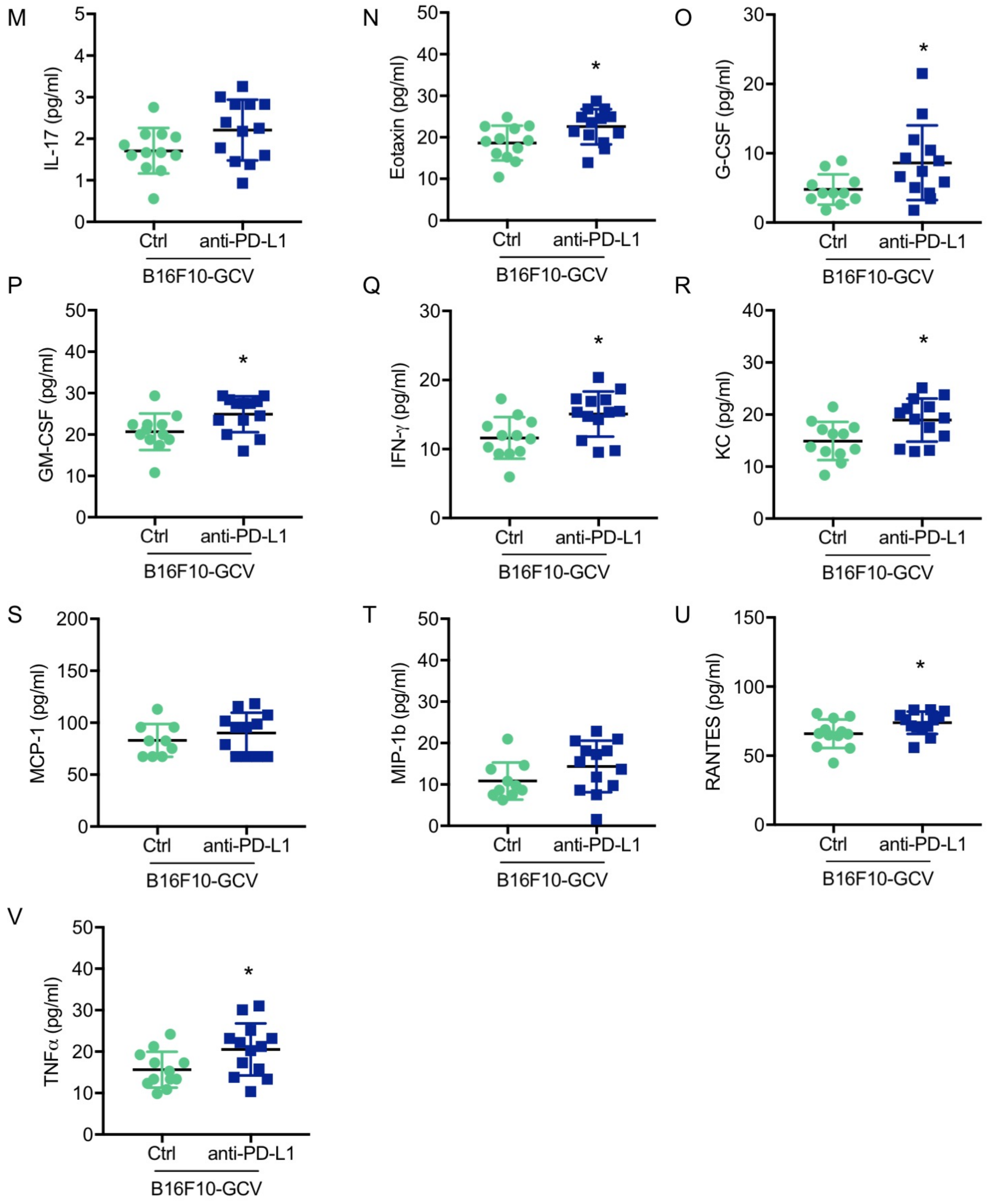


**Supplementary Figure 3** Protein expression in LV tissue of anti-PD-L1 treatment in B16F10-GCV mice. Dot plots summarizing (A) IL-1a, (B) IL-1b, (C) IL-2, (D) IL-3, (E) IL-4, (F) IL-5, (G) IL-6, (H) IL-9, (I) IL-10, (J) IL-12(p40), (K) IL-12(p70), (L) IL-13, (M) IL-17, (N) Eotaxin, (O) G-CSF, (P) GM-CSF, (Q) IFN-γ, (R) KC, (S) MCP-1, (T) MIP-1a, (U) MIP-1b, (V) RANTES and (W) TNFα protein levels (pg/ml) detected in LV tissue lysates of anti-PD-L1 (N=13) or isotype Ctrl (N=12) treated B16F10-GCV mice by Bio-Rad Multiplex Assay. (B-J, L-W) Gaussian distributed data were presented as mean±SD and (A, K) not normally distributed data were presented as median and IQR, *P<0.05 vs B16F10-GCV isoptype Ctrl, unpaired two-tailed Student's *t* test or Mann-Whitney *U* test.

**Supplementary Figure 4**

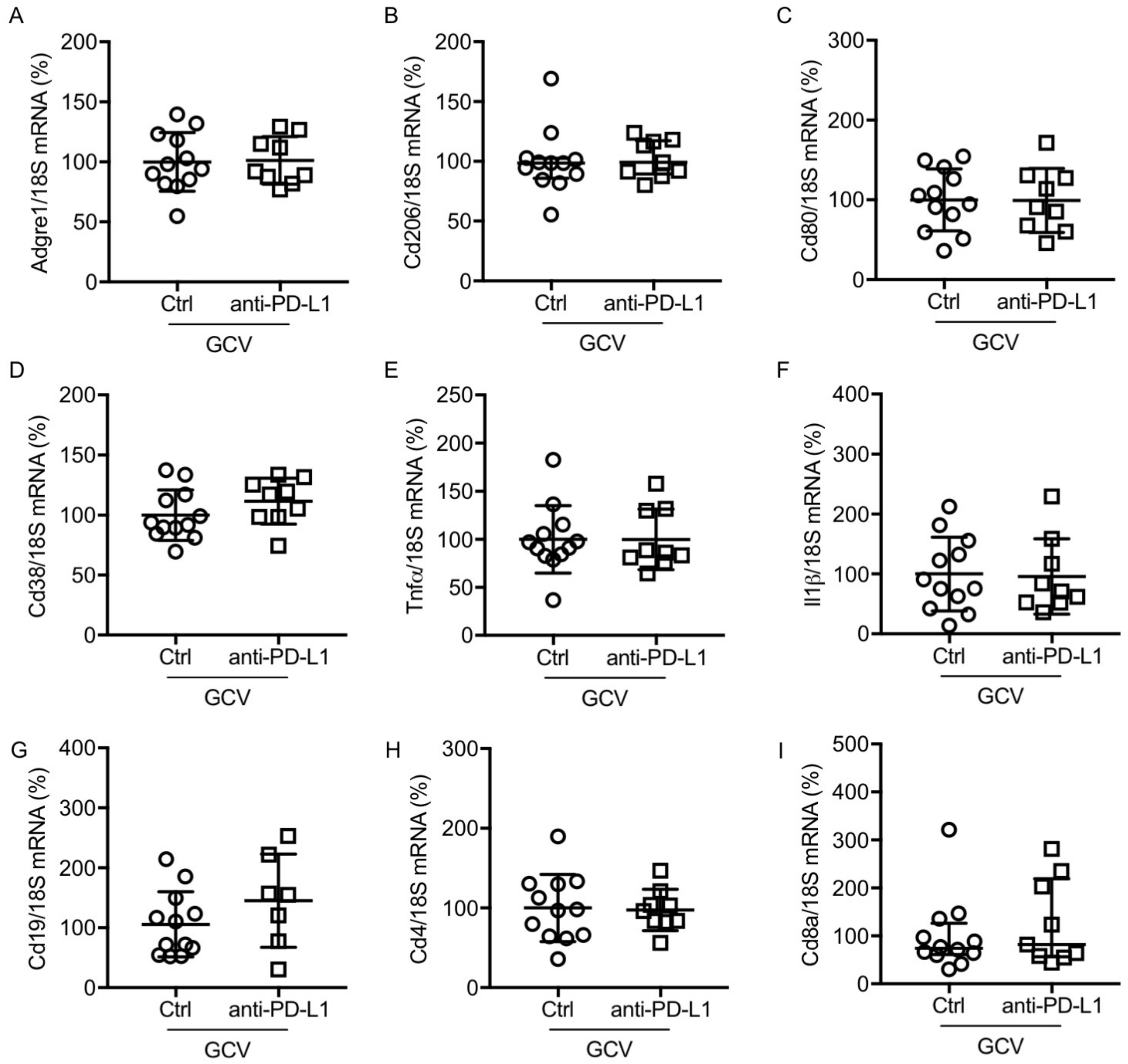


**Supplementary Figure 4** Inflammatory gene expression of anti-PD-L1 treatment in GCV mice. Dot plots summarizing (A) *Adgre1*, (B) *Cd206*, (C) *Cd80*, (D) *Cd38*, (E) *Tnfα,* (F) *Il-1β,* (G) *Cd19,* (H) *Cd4* and (I) *Cd8a* mRNA levels normalised to 18S RNA analysed by qRT-PCR in GCV LVs treated with anti-PD-L1 (N=9; (except (G) N=7) or isotype Ctrl (N=12). (A, C-F) Gaussian distributed data were presented as mean±SD and (B) not normally distributed data were presented as median and IQR, not significant (n.s.), unpaired two-tailed Student's *t* test or Mann-Whitney *U* test.

**Supplementary Figure 5**

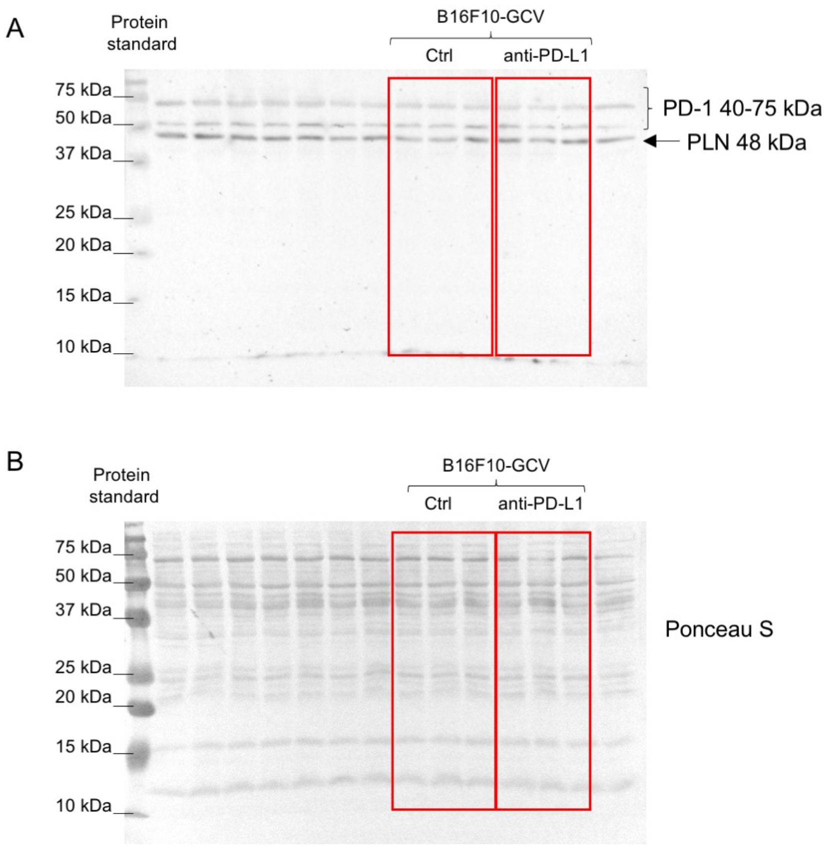


**Supplementary Figure 5** Uncropped full length western blots. Whole gel images of representative western blots shown in Fig. 2K of (A) PD-1 and (B) corresponding Ponceau S staining from LV tissue of B16F10-GCV mice treated with isotype Ctrl or anti-PD-L1. Red boxes mark the cropped lanes, which are presented in the Fig. 2K.

## Supplementary Figure 6

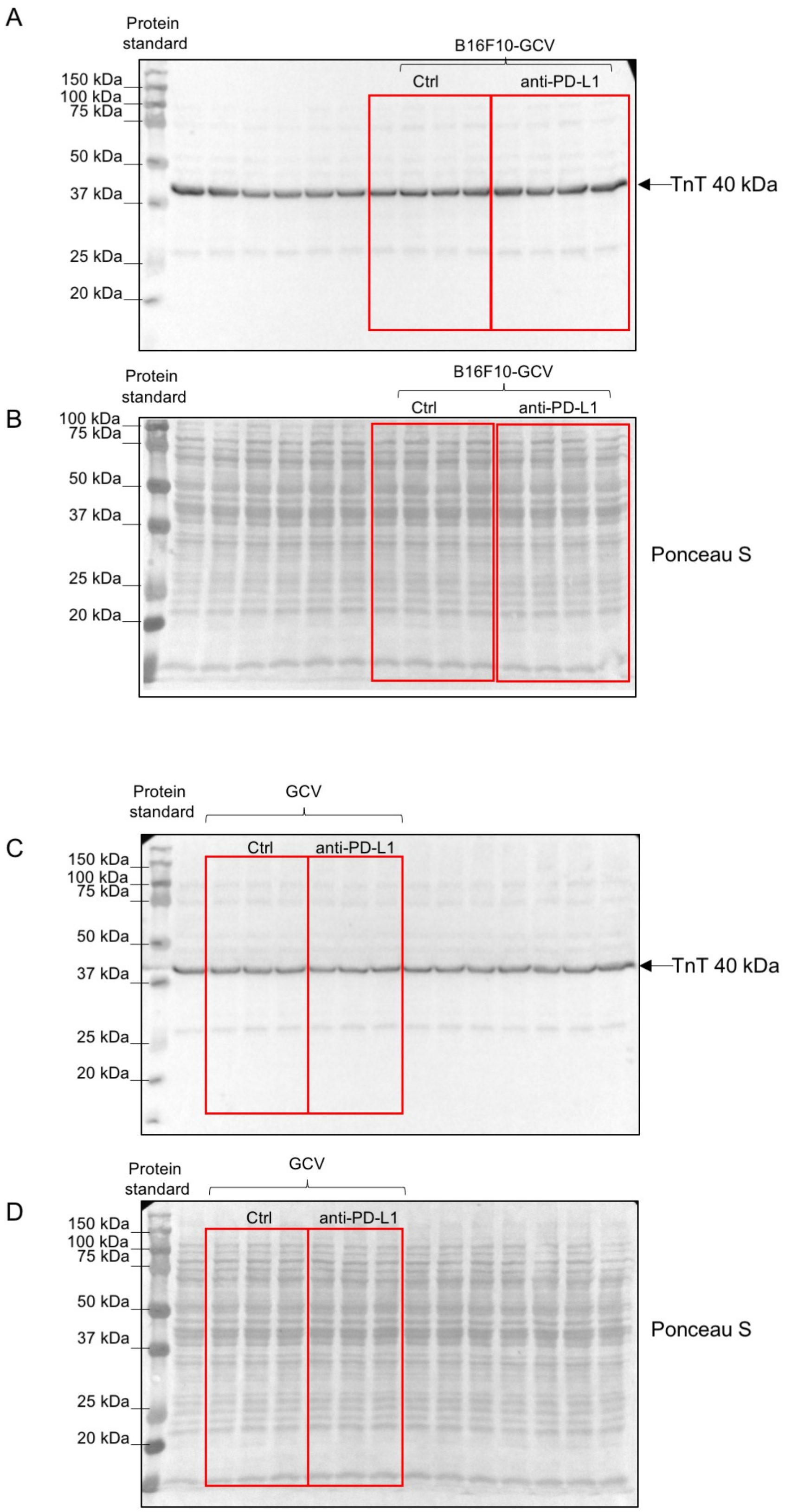

**Supplementary Figure 6** Uncropped full length western blots. Whole gel images of representative western blots shown in Fig. 3D of (A) TnT and (B) corresponding Ponceau S staining from LV tissue of B16F10-GCV mice treated with isotype Ctrl or anti-PD-L1, and in SFig. 1H (C) TnT and (D) corresponding Ponceau S staining from LV tissue GCV mice treated with isotype Ctrl or anti-PD-L1. Red boxes mark the cropped lanes, which are presented in the Fig. 3D and SFig 1H.

## Supplementary Figure 7

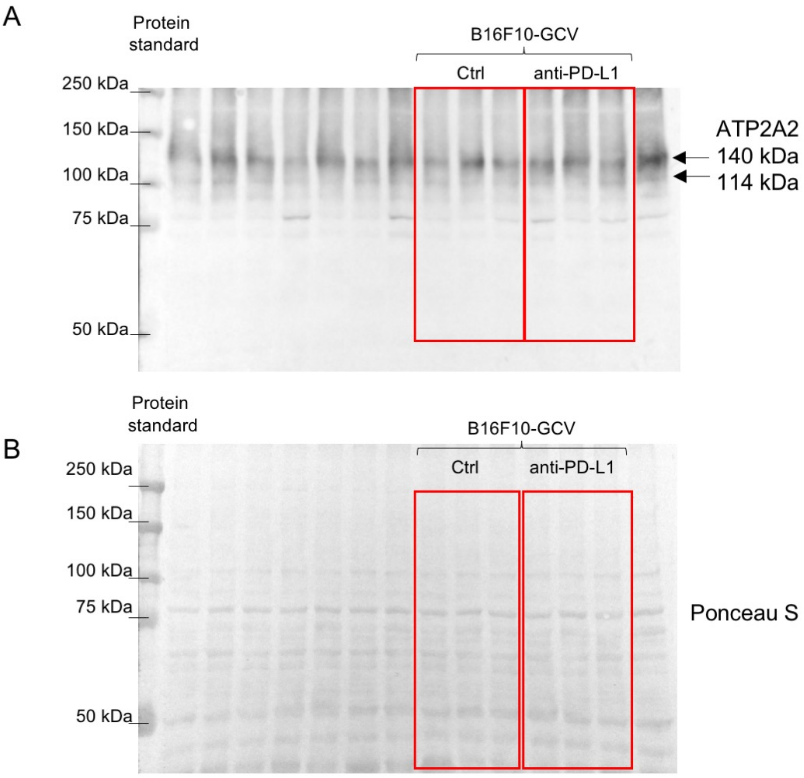


**Supplementary Figure 7** Uncropped full length western blots. Whole gel images of representative western blots shown in Fig. 3K of (A) ATP2A2 and (B) corresponding Ponceau S staining from LV tissue of B16F10-GCV mice treated with isotype Ctrl or anti-PD-L1. Red boxes mark the cropped lanes, which are presented in the Fig. 3K.

**Supplementary Figure 8**

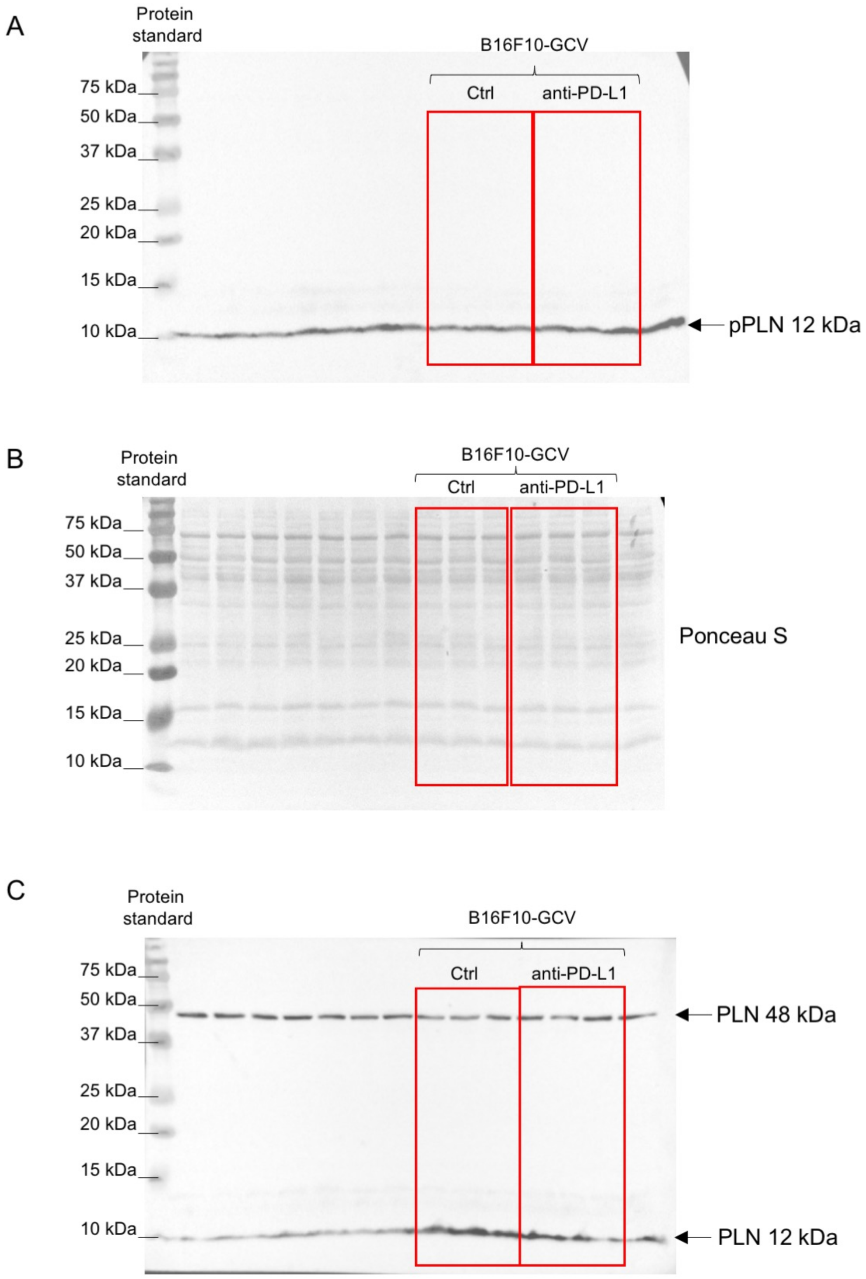


**Supplementary Figure 8** Uncropped full length western blots. Whole gel images of representative western blots shown in SFig. 2A of (A) pPLN, (B) corresponding Ponceau S staining and (C) PLN from LV tissue of B16F10-GCV mice treated with isotype Ctrl or anti-PD-L1. Red boxes mark the cropped lanes, which are presented in the SFig. 2A.

## Supplementary Figure 9

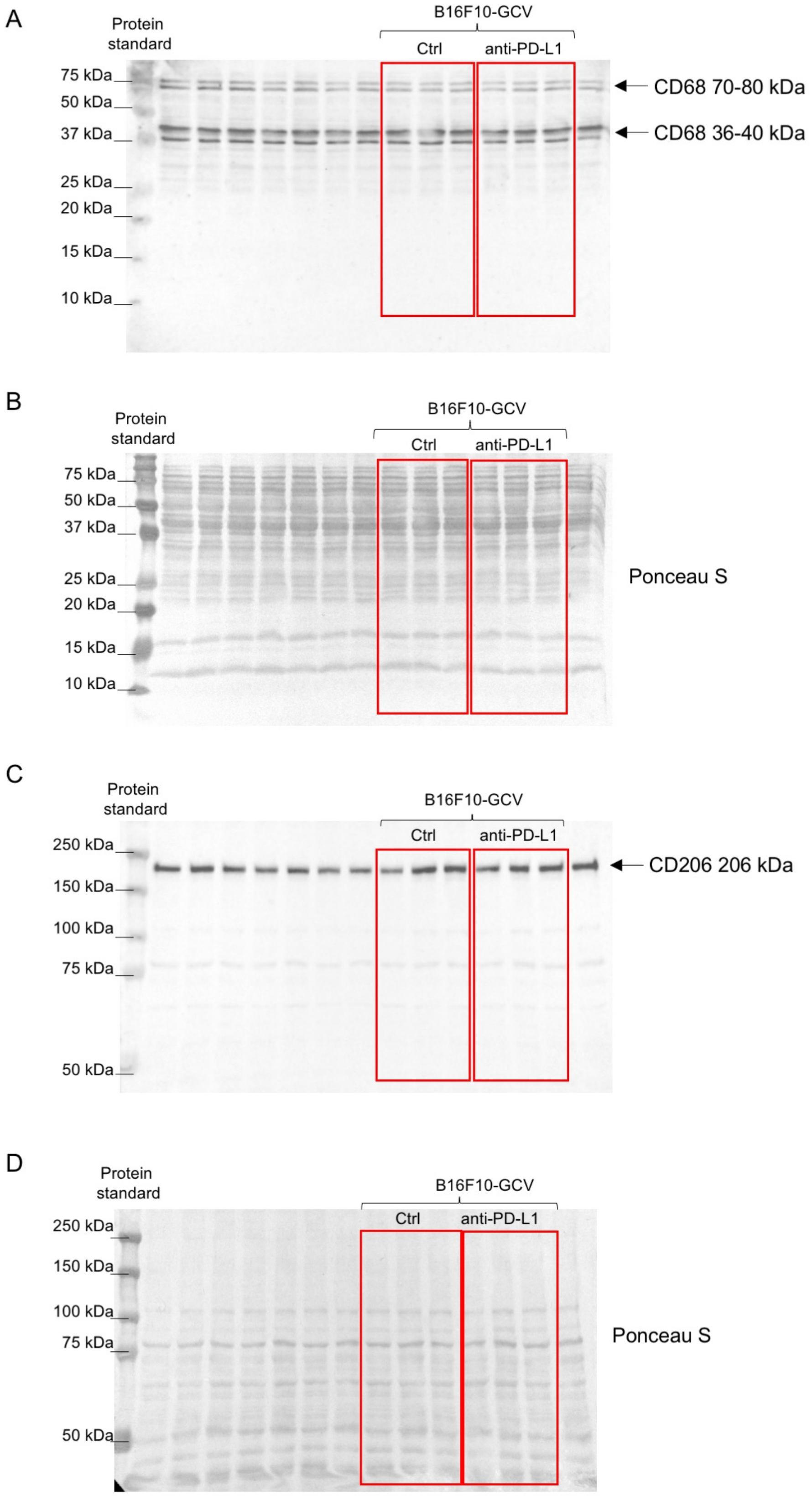

E

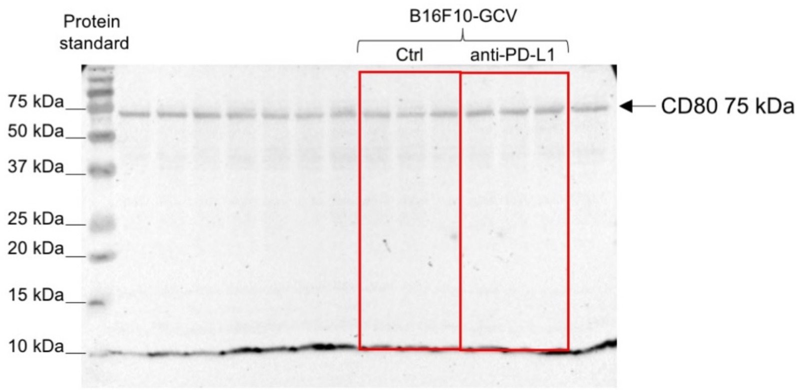


**Supplementary Figure 9** Uncropped full length western blots. Whole gel images of representative western blots shown in Fig. 8A-C of (A) CD68, (B) corresponding Ponceau S staining, (C) CD206, (D) corresponding Ponceau S staining and (E) CD80 from LV tissue of B16F10-GCV mice treated with isotype Ctrl or anti-PD-L1. Red boxes mark the cropped lanes, which are presented in the Fig. 8A-C.
